# Naphthoquinone amino acid derivatives, synthesis and biological activity as proteasome inhibitors

**DOI:** 10.1080/14756366.2017.1334649

**Published:** 2017-06-28

**Authors:** Mauro Marastoni, Claudio Trapella, Alessandra Scotti, Anna Fantinati, Valeria Ferretti, Erika Marzola, Gallerani Eleonora, Riccardo Gavioli, Delia Preti

**Affiliations:** a Department of Chemical and Pharmaceutical Sciences, University of Ferrara, Ferrara, Italy;; b Department of Life Sciences and Biotechnology, University of Ferrara, Ferrara, Italy

**Keywords:** Proteasome, naphthoquinone, amino acid derivatives, post-acidic inhibition

## Abstract

The ubiquitin-proteasome system has been largely investigated for its key role in protein degradation mechanisms that regulate both apoptosis and cell division. Because of their antitumour activity, different classes of proteasome inhibitors have been identified to date. Some of these compounds are currently employed in the clinical treatment of several types of cancer among which multiple myeloma. Here, we describe the design, chemistry, biological activity and modelling studies of a large series of amino acid derivatives linked to a naphthoquinone pharmacophoric group through variable spacers. Some analogues showed interesting inhibitory potency for the β1 and β5 subunits of the proteasome with IC_50_ values in the sub-µm range.

## Introduction

The proteasome enzymatic complex is widely involved in the cytosolic and nuclear catabolism of most proteins behaving as a protease with multiple catalytic sites^1^. The proteolytic action of proteasome allows the elimination of impaired proteins and results in the production of short-chain peptides that can be exposed by the MHC complexes^2^. The ubiquitin-proteasome system has been largely investigated for its key role in protein degradation mechanisms that regulate both apoptosis and cell division^3^^,^^4^. According to this system, the degradation of proteins is the result of their conjugation with multiple ubiquitin units that allow the recognition by the 26 S proteasome. The 26 S proteasome is composed of multiple subunits and is characterised by a central 20 S core and two distal regions (19 S) displaying regulatory activity. In the 20 S core, two external heptameric rings of α-subunits enclose two central heptameric rings of β-subunits. The 20 S core exerts three typical catalytic activities that are specifically located in the β1 (the peptidyl glutamyl peptide hydrolysing activity, PGPH), β2 (the trypsin-like activity, T-L) and β5 (the chymotrypsin-like, ChT-L) subunits^5^. The three catalytic β subunits have a slightly different substrate specificity with a common mechanism of proteolysis through an N-terminal threonine-dependent nucleophilic attack. Most of the cellular proteins undergo degradation through this pathway that affects several processes such as cell division, apoptosis or repair of DNA damages. As a consequence, any alteration of this system may result in important pathologies, including cancer.

Proteasome regulation by exogenous molecules able to manipulate cellular activities has been receiving increasing attention^6^. Inhibitors of this multicatalytic complex are potential drugs suitable in various therapeutic applications, therefore, natural and synthetic molecules have been studied as 20 S catalytic subunits inhibitors^7–16^. The first-generation proteasome inhibitor (PI) bortezomib is currently employed as an anti-cancer drug, although its effectiveness seems to be restricted to a limited number of cancers^17^^,^^18^. The FDA-approved carfilzomib^19^ and ixazomib^20^ along with oprozomib^21^, currently in advanced clinical trials, are examples of second-generation irreversible PIs with a peptide structure.

**Figure 1.**
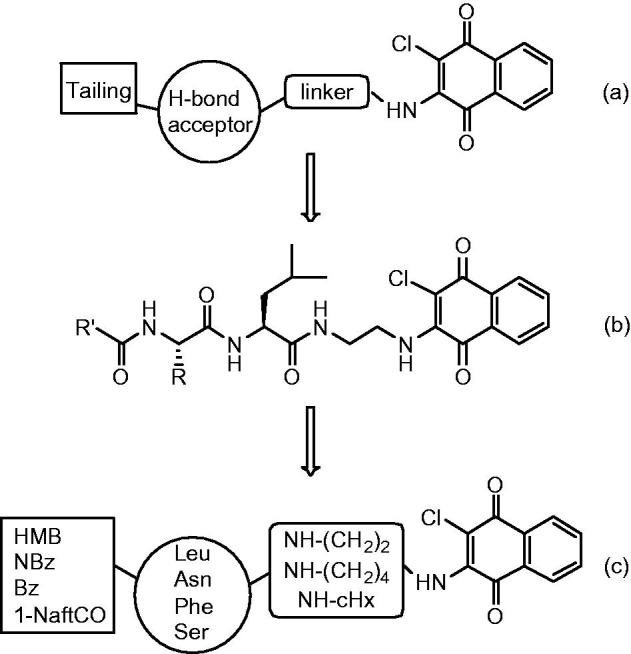
(a) Schematic structure of non-peptide inhibitors bearing the 2-cloronaphthoquinonic unit. (b) The general structure of dipeptide derivatives with a 2-chloronaphthoquinone group. (c) The generic structure of the new amino acid derivatives linked to the 2-chloronaphthoquinone group.

In the last years, we developed several classes of peptide-based PIs having different pharmacophoric units such as electrophilic groups potentially able to interact with the catalytic threonine^22–24^. We have recently investigated a new series of dipeptide-based derivatives bearing at the C-terminal a 2-chloronaphthoquinone pharmacophoric group (structure **b** in Figure 1)^25^. Some compounds of this series have been shown to inhibit the post-acidic-like and the ChT-L active sites of the proteasome in the µm range. The compound named PI-083(NSC-45382), bearing the 2-cloronaphthoquinonic unit, and other non-peptide analogues (general structure **a** in Figure 1), are known to express a good inhibition against chymotryptic activity of the proteasome and the capacity to selectively inhibit tumour cell proliferation^26–28^.

Herein, we describe the synthesis, the *in vitro* biological evaluation of proteasome inhibition and modelling studies of a new series of amino acid derivatives linked through the α-carboxylic function to the 2-chloronaphthoquinone pharmacophoric group (general structure **c** in Figure 1). The 2-chloronaphthoquinone (ClNafQ), a potential electrophilic substrate for the catalytic threonine, is linked to the selected residues by a diamine alkyl spacers. Studies regarding the non-peptide PI-083 and its analogues, in addition to our docking analysis with the previous dipeptidic derivatives, suggest the potential interaction of the γ-hydroxyl group of catalytic threonine with the 2-chloronaphthoquinone unit. The L-amino acids (Leu, Asn, Phe, Ser) were selected for their different physicochemical features. The chloronaphthoquinone pharmacophore is linked to the carboxylic group of the central residue by ethylenediamine (compounds **1**–**16**), butylenediamine (**17–32**) and cyclohexyldiamine (**33**–**48**) spacers having different length and flexibility (see Table 1 for the detailed structures). Finally, the α-amino group is functionalised with 2-methyl-3-hydroxybenzoyl (HMB), p-nitrobenzoyl (NBz), benzoyl (Bz) or 1-naphthoyl (1-NaftCO) aromatic groups having variable electronic and steric peculiarity.

**Table 1.** Inhibition of the proteasome subunits by the synthesised compounds.


Compd	R'	Xaa	R	**IC_**50**_ (μm)**^a^**T-L**	**IC_**50**_ (μm)**^a^**ChT-L**	**IC_**50**_ (μm)**^a^**PGPH**
**1**	HMB	Leu	–(CH_2_)_2_−	77.44 ± 6.51	>100	1.21 ± 0.08
**2**	NBz	Leu	–(CH_2_)_2_−	65.32 ± 5.91	>100	2.35 ± 0.21
**3**	Bz	Leu	–(CH_2_)_2_−	58.43 ± 5.13	>100	2.61 ± 0.23
**4**	1-NaftCO	Leu	–(CH_2_)_2_−	21.08 ± 1.85	0.82 ± 0.08	0.91 ± 0.07
**5**	HMB	Asn	–(CH_2_)_2_−	68.35 ± 5.14	8.47 ± 0.75	6.18 ± 0.55
**6**	NBz	Asn	–(CH_2_)_2_−	17.23 ± 1.09	0.92 ± 0.09	1.19 ± 0.13
**7**	Bz	Asn	–(CH_2_)_2_−	49.66 ± 3.87	7.99 ± 0.67	11.23 ± 0.98
**8**	1-NaftCO	Asn	–(CH_2_)_2_−	35.23 ± 2.77	76.25 ± 6.82	10.01 ± 1.27
**9**	HMB	Phe	–(CH_2_)_2_−	>100	0.85 ± 0.07	0.88 ± 0.08
**10**	NBz	Phe	–(CH_2_)_2_−	35.40 ± 2.91	0.77 ± 0.06	0.24 ± 0.3
**11**	Bz	Phe	–(CH_2_)_2_−	53.18 ± 6.70	6.22 ± 0.48	1.05 ± 0.09
**12**	1-NaftCO	Phe	–(CH_2_)_2_−	49.75 ± 3.97	9.14 ± 1.02	35.76 ± 4.03
**13**	HMB	Ser	–(CH_2_)_2_−	>100	>100	91.52 ± 7.69
**14**	NBz	Ser	–(CH_2_)_2_−	63.36 ± 7.01	83.47 ± 8.03	78.66 ± 8.14
**15**	Bz	Ser	–(CH_2_)_2_−	53.82 ± 4.88	24.11 ± 1.98	5.78 ± 4.35
**16**	1-NaftCO	Ser	–(CH_2_)_2_−	>100	>100	>100
**17**	HMB	Leu	–(CH_2_)_4_−	>100	11.50 ± 1.02	7.82 ± 0.71
**18**	NBz	Leu	–(CH_2_)_4_−	>100	8.40 ± 0.72	10.54 ± 0.82
**19**	Bz	Leu	–(CH_2_)_4_−	>100	10.12 ± 0.70	2.41 ± 0.14
**20**	1-NaftCO	Leu	–(CH_2_)_4_−	>100	49.30 ± 5.02	45.35 ± 4.03
**21**	HMB	Asn	–(CH_2_)_4_−	>100	65.38 ± 6.50	9.52 ± 1.09
**22**	NBz	Asn	–(CH_2_)_4_−	>100	28.52 ± 3.05	>100
**23**	Bz	Asn	–(CH_2_)_4_−	>100	24.19 ± 2.05	>100
**24**	1-NaftCO	Asn	–(CH_2_)_4_−	>100	78.34 ± 6.95	>100
**25**	HMB	Phe	–(CH_2_)_4_−	>100	17.50 ± 1.07	8.82 ± 0.71
**26**	NBz	Phe	–(CH_2_)_4_−	>100	9.10 ± 0.6	10.54 ± 0.82
**27**	Bz	Phe	–(CH_2_)_4_−	>100	10.12 ± 0.70	5.43 ± 0.44
**28**	1-NaftCO	Phe	–(CH_2_)_4_−	79.71 ± 6.97	9.30 ± 1.02	25.65 ± 3.03
**29**	HMB	Ser	–(CH_2_)_4_−	80.24 ± 6.91	45.68 ± 2.59	61.52 ± 4.99
**30**	NBz	Ser	–(CH_2_)_4_−	83.26 ± 7.21	88.74 ± 8.03	83.66 ± 8.16
**31**	Bz	Ser	–(CH_2_)_4_−	>100	84.11 ± 6.08	3.55 ± 1.65
**32**	1-NaftCO	Ser	–(CH_2_)_4_−	60.75 ± 4.88	>100	83.62 ± 5.48
**33**	HMB	Leu	–cHx-	>100	>100	>100
**34**	NBz	Leu	–cHx-	>100	42.10 ± 5.02	>100
**35**	Bz	Leu	–cHx-	>100	23.12 ± 1.70	>100
**36**	1-NaftCO	Leu	–cHx-	>100	59.30 ± 6.42	73.67 ± 1.45
**37**	HMB	Asn	–cHx-	>100	>100	>100
**38**	NBz	Asn	–cHx-	>100	>100	>100
**39**	Bz	Asn	–cHx-	>100	>100	>100
**40**	1-NaftCO	Asn	–cHx-	>100	>100	>100
**41**	HMB	Phe	–cHx-	>100	>100	>100
**42**	NBz	Phe	–cHx-	>100	82.10 ± 6.52	>100
**43**	Bz	Phe	–cHx-	>100	73.31 ± 3.90	>100
**44**	1-NaftCO	Phe	–cHx-	>100	>100	83.37 ± 4.58
**45**	HMB	Ser	–cHx-	>100	>100	>100
**46**	NBz	Ser	–cHx-	>100	>100	5.78 ± 0.62
**47**	Bz	Ser	–cHx-	>100	>100	3.42 ± 0.51
**48**	1-NaftCO	Ser	–cHx-	>100	>100	11.71 ± 1.25
**MG132**				1.04 ± 0.092	0.0018 ± 0.00022	>10

a The values reported are the mean ± SEM of three independent determinations.

## Methods and materials

### Chemistry-general

Amino acids, amino acid derivatives and chemicals were purchased from Bachem, Novabiochem, and Fluka (Switzerland). Crude products were purified by preparative reversed-phase HPLC using a Waters Delta Prep 3000 system with a Jupiter column C_18_ (250 × 30 mm, 300 Å, 15 µ spherical particle size). The column was perfused at a flow rate of 20 ml/min, with a mobile phase-containing solvent A (10%, v/v, acetonitrile in 0.1% TFA), and a linear gradient from 0% to 100% of solvent B (60%, v/v, acetonitrile in 0.1% TFA); 30 min was the time adopted for elution of the compounds. HPLC analysis was performed using a Beckman System Gold with a Luna C_18_ column (4.6 × 100 mm, 3 µ particle size). Analytical determination and retention time (T_r_) of the peptides were assayed via HPLC conditions in the above solvent system (solvents A and B), programmed at flow rates of 0.5 ml/min, using the following linear gradients: (a) from 0% to 90% B for 25 min and (b) from 30% to 100% B for 25 min. No naphthoquinone derivative showed more than 1% impurity when monitored at 220 and 254 nm. The molecular weights of the compounds were determined by electrospray ionisation (ESI) (MICROMASS ZMD 2000), and the values are expressed as [M + H]^+^. TLC was performed on pre-coated plates of silica gel F254 (Merck, Darmstadt, Germany), exploiting the following solvent systems: (c) AcOEt/*n*-hexane (1:1, v/v), (d) CH_2_Cl_2_/methanol (9.5:0.5, v/v), (e) CH_2_CL_2_/methanol (9:1, v/v) and (f) CH_2_CL_2_/methanol/toluene (17:2:1, v/v/v). Ninhydrin (1%) or chlorine iodine spray reagents were employed to detect the peptides. Melting points were determined by a Kofler apparatus and are uncorrected. Optical rotations were determined by a Perkin–Elmer 141 polarimeter with a 10-cm water-jacketed cell. ^1^H NMR spectroscopy was obtained using a Varian 400 MHz spectrometer. All the assayed compounds described in this manuscript were at least 95% pure as judged by HPLC and NMR.

### Synthesis

**Scheme 1.**
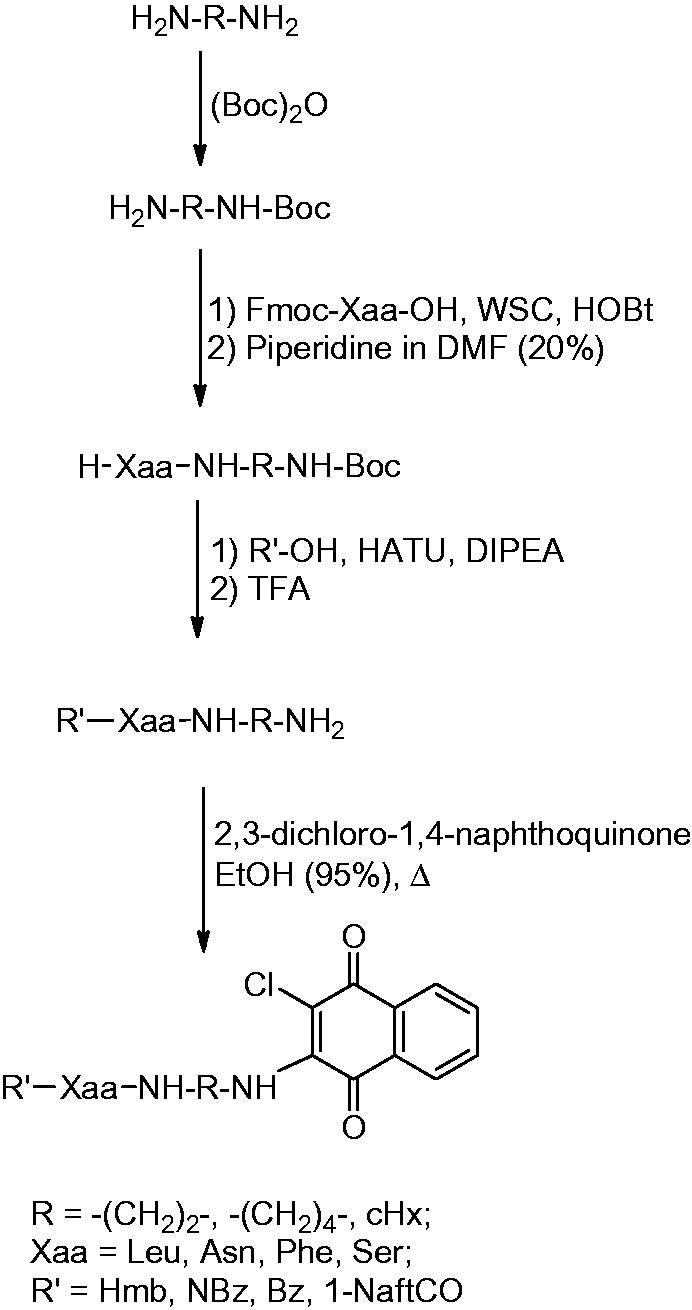
Synthesis of naphthoquinone amino acid derivatives.

The synthesis applied for the preparation of compounds **1**–**48** is reported in Scheme 1. This synthetic strategy allowed us to obtain the compounds with the least possible number of steps. The entire synthesis was performed in solution wherein the L-amino acids were suitably protected at the Nα and linked to the selected diamine spacers at the carboxylic group. The alkyldiamine linkers were protected at one of the amine functionality with the Boc group. Subsequently, the Fmoc-protected amino acids were condensed at the mono-protected diaminic spacers using 1-ethyl-3-(3-dimethylaminopropyl)-carbodiimmide (WSC) and N-hydroxybenzotriazole (HOBt) as acylating agents. After treatment with piperidine to remove the fluorenylmethoxycarbonyl (Fmoc), the Nα functionalisation was carried out by acylation with 3-hydroxy-2-methyl-benzoic acid, 4-nitrobenzoic acid, benzoic acid or alpha-naphthoic acid using 2-(1H-9-azabenzotriazole-1-yl)-1,1,3,3-tetramethyl-aminium hexafluorophosphate (HATU) as a coupling agent. After the Boc removal via TFA treatment, we proceeded to the final reaction with 2,3-dichloro-naphthoquinone that takes place in a solution of 95% EtOH, in presence of *N*-methyl-morpholine. All products, after purification by RP-HPLC, were analysed by mass spectrometry and NMR. The analytical data of the compounds are reported in the supporting material.

#### General synthetic procedures

- *Condensation with Fmoc-amino acids.* The carboxylic component (1 mmol) was dissolved DMF (10 ml) and, after cooling at 0 °C, WSC (1.1 mmol), HOBt (1.1 mmol) and the amine component (1.1 mmol) were added. The reaction mixture was stirred for 1 h at 0 °C then overnight at room temperature. The solvent was evaporated to give a residue that was suspended with EtOAc and washed successively with 10% citric acid (10 ml), 5% NaHCO_3_ (10 ml) and again with brine (10 ml). The organic phase was dried with Na_2_SO_4_, filtered and evaporated to furnish the desired products that were used without further purification.
- *Condensation with 2-methyl-3-hydroxybenzoic acid, p-nitrobenzoic acid, benzoic acid or α-naphthoic acid.* The carboxylic component (1 mmol) was dissolved DMF (6 ml) and HATU (1mmol) and DIPEA (1 mmol) were added. Then a solution of the appropriate amine (1 mmol) and TEA (1 mmol) in DMF (6 ml) was added. The mixture was stirred overnight at room temperature. The solvent was evaporated to obtain a residue that was suspended with EtOAc. The organic phase was washed successively with 10% citric acid (2 × 5 ml), 5% NaHCO_3_ (2 × 5 ml) and again with brine (2 × 5 ml). The organic phase was dried with Na_2_SO_4_, filtered and evaporated to give a solid residue that was crystallised (Et_2_O) and collected after centrifugation.
- *Fmoc Removal.* The Fmoc protection was removed by treatment at room temperature with a 20% piperidine solution in DMF for 1 h. The solvent was evaporated and the desired products were precipitated with ethyl ether, then separated by centrifugation and collected.
- *Boc Removal.* The Boc protection was removed by treatment with 90% aqueous TFA (1 ml for 1 mmol of the Boc-protected compound) for 1 h. After evaporation of the solvent, the residue was triturated with ethyl ether and separated by centrifugation.
- *Condensation with 2,3-dichloro-1,4-naphthoquinone.* The amine component (0.3 mmol) was dissolved in 95% EtOH (15 ml) then N-methyl-morpholine (0.3 mmol) and 2,3-dichloro-1,4-naphthoquinone (0.6 mmol) were added. The mixture was heated at 115 °C for 3 d under stirring. After evaporation of the solvent, the residue was triturated with ethyl ether and separated by centrifugation.

#### Preparation of Boc-ethylene/butylene/trans-cyclohexyldiamine

The diamine (10 mmol) was dissolved in a mixture of t-ButOH/H_2_O (2:1, 20 ml) then (Boc)_2 _O (7 mmol) was added and the reaction was stirred for 2 h at room temperature. Water (20 ml) was added and the aqueous phase was extracted with n-pentane (2 × 10 ml). After separation, the aqueous phase was further extracted with EtOAc (2 × 50 ml) and the latter organic phase was dried with anhydrous Na_2_SO_4_ and evaporated to yield the desired compounds that were employed without further purification.

*Boc-ethylenediamine.* Colourless oil, yield 85%.^1^H NMR (CDCl_3_) δ5.98 (bs, 1H), 3.08 (m, 2H), 2.69 (m, 2H), 1.75 (bs, 2H), 1.39 (s, 9H); MS (M + H^+^) 161.20; HPLC (T_r_) 6.54 min. Spectroscopic data are consistent with those previously reported^25^.

*Boc-butylenediamine.* Colourless oil, yield 75%. ^1^H NMR (CDCl_3_) δ 4.70 (bs, 1H), 3.14 (m, 2H), 2.68 (t, 2H, J = 6.7), 1.68 (bs, 2H), 1.50–1.45 (m, 4H), 1.48 (s, 9H). MS (M + H^+^) 189.22; HPLC (T_r_) 7.24 min. Spectroscopic data are consistent with those previously reported^29^.

*Boc-trans-cyclohexyldiamine.* White solid, yield 96%. ^1^H NMR (CDCl_3_): δ 4.91–5.12 (bs, 1H), 3.31–3.41 (bs, 1H), 2.57–2.68 (m, 1H), 1.90–2.03 (bs, 2H), 1.87–1.97 (m, 4H), 1.44 (s, 9H), 1.10–1.24 (m, 4H). MS (M + H^+^) 214.26; HPLC (Tr) 7.56 min. Spectroscopic data are consistent with those previously reported^30^.

#### Preparation of H-Xaa-NH-R-NH-boc

The intermediates with general structure Fmoc-Xaa-NH-R-NH-Boc were first prepared by acylation of the appropriate Boc-protected diamine with Fmoc-Xaa-OH following the general procedure (a). Fmoc-protected derivatives were then treated according to the general procedure for Fmoc removal (c) to give the desired H-Xaa-NH-R-NH-Boc intermediates.

*H-Leu-NH-(CH_2_)_2_-NH-Boc.* White solid, yield 77%; ^1^H NMR, (CDCl_3_) δ: 3.49–3.41 (m, 2H), 3.18–3.14 (m, 1H), 3.05–3.00 (m, 1H), 2.90–2.88 (m, 1H), 1.78–1.76 (m, 1H), 1.39 (s, 9H), 1.36–1.32 (m, 2H), 0.94 (d, 3H, *J* = 7.4 Hz), 0.89 (d, 3H, *J* = 7.4 Hz); MS (M + H^+^) 274.31.

*H-Asn-NH-(CH_2_)_2_-NH-Boc.* White solid, yield 67%; ^1^H NMR, (CDCl_3_) δ: 7.82 (bs, 1H), 5.11 (s, 2H), 3.79–3.76 (m, 1H), 3.58–3.54 (m, 2H), 3.47–3.42 (m, 2H), 3.12–2.90 (m, 1H), 2.85–2.72 (m, 1H), 1.45 (s, 9H);MS (M + H^+^) 275.32.

*H-Phe-NH-(CH_2_)_2_-NH-Boc.* White solid, yield 88%; ^1^H NMR, (CDCl_3_) δ: 7.76 (bs, 1H), 7.44–7.42 (m, 2H), 7.32–7.29 (m, 3H), 5.13 (s, 2H), 3.91–3.88 (m, 1H), 3.56–3.52 (m, 2H), 3.46–3.45 (m, 2H), 3.44–3.42 (m, 1H), 3.20–3.18 (m, 1H), 1.44 (s, 9H);MS (M + H^+^) 308.30.

*H-Ser-NH-(CH_2_)_2_-NH-Boc.* White solid, yield 67%; ^1^H NMR, (CDCl_3_) δ: 7.74 (bs, 1H), 5.13 (bs, 2H), 4.18–4.16 (m, 1H), 3.92–3.90 (m, 1H), 3.66–3.64 (m, 1H), 3.53–3.50 (m, 2H), 3.48–3.46 (m, 2H), 1.38 (s, 9H);MS (M + H^+^) 248.28.

*H-Leu-NH-(CH_2_)_4_-NH-Boc.* White solid, yield 77%; ^1^H NMR, (CDCl_3_) δ: 8.00 (bs, 1H), 5.40 (bs, 2H), 3.49–3.41 (m, 5H), 1.78–1.76 (m, 1H), 1.55–1.54 (m, 2H), 1.51–1.49 (m, 2H), 1.43 (s, 9H), 1.34–1.33 (m, 2H), 0.93 (d, 3H, *J* = 7.4 Hz), 0.90 (d, 3H, *J* = 7.4 Hz);MS (M + H^+^) 302.24.

*H-Asn-NH-(CH_2_)_4_-NH-Boc.* White solid, yield 87%; ^1^H NMR, (CDCl_3_) δ: 7.79 (bs, 1H), 5.33 (bs, 2H), 3.79–3.77 (m, 1H), 3.55–3.53 (m, 2H), 3.44–3.42 (m, 2H), 2.91–2.89 (m, 1H), 2.87–2.86 (m, 1H), 1.54–1.53 (m, 2H), 1.50–1.49 (m, 2H), 1.43 (s, 9H);MS (M + H^+^) 303.27.

*H-Phe-NH-(CH_2_)_4_-NH-Boc.* White solid, yield 64%;^1^H NMR, (CDCl_3_) δ: 7.99 (bs, 1H), 7.44–7.42 (m, 2H), 7.31–7.29 (m, 3H), 5.22 (bs, 2H), 3.89–3.87 (m, 1H), 3.56–3.53 (m, 1H), 3.22–3.18 (m, 5H), 1.52–1.50 (m, 2H), 1.49–1.47 (m, 2H), 1.42 (s, 9H);MS (M + H^+^) 336.18.

*H-Ser-NH-(CH_2_)_4_-NH-Boc.* White solid, yield 77%; ^1^H NMR, (CDCl_3_) δ: 8.03 (bs, 1H), 5.20 (bs, 2H), 4.17–4.15 (m, 1H), 3.90–3.89 (m, 1H), 3.64–3.62 (m, 1H), 3.50–3.49 (m, 2H), 3.48–3.47 (m, 2H), 1.54–1.53 (m, 2H), 1.51–1.49 (m, 2H), 1.43 (s, 9H);MS (M + H^+^) 276.29.

*H-Leu-NH-cHx-NH-Boc.* White solid, yield 77%; ^1^H NMR, (CDCl_3_) δ: 7.64 (bs, 1H), 5.44 (bs, 2H), 3.55–3.54 (m, 1H), 3.52–3.51 (m, 1H), 3.35–3.33 (m, 1H), 1.77–1.75 (m, 1H), 1.70–1.69 (m, 4H), 1.54–1.53 (m, 4H), 1.39 (s, 9H), 1.34–1.32 (m, 2H), 0.94 (d, 3H, *J* = 7.4 Hz), 0.89 (d, 3H, *J* = 7.4 Hz); MS (M + H^+^) 328.22.

*H-Asn-NH-cHx-NH-Boc.* White solid, yield 87%; ^1^H NMR, (CDCl_3_) δ: 8.26 (bs, 1H), 7.24 (bs, 2H), 5.54 (bs, 2H), 3.78–3.76 (m, 1H), 3.55–3.53 (m, 2H), 2.90–2.88 (m, 1H), 2.85–2.83 (m, 1H), 1.77–1.73 (m, 4H), 1.50–1.47 (m, 4H), 1.38 (s, 9H);MS (M + H^+^) 329.32.

*H-Phe-NH-cHx-NH-Boc.* White solid, yield 56%; ^1^H NMR, (CDCl_3_) δ: 8.44 (bs, 1H), 7.42–7.40 (m, 2H), 7.31–7.29 (m, 3H), 5.44 (bs, 2H), 3.89–3.87 (m, 1H), 3.58–3.56 (m, 1H), 3.54–3.53 (m, 2H), 3.19–3.18 (m, 1H), 1.78–1.73 (m, 4H), 1.49–1.44 (m, 4H), 1.38 (s, 9H);MS (M + H^+^) 362.15.

*H-Ser-NH-cHx-NH-Boc.* White solid, yield 57%; ^1^H NMR, (CDCl_3_) δ: 8.15 (bs, 1H), 5.44 (bs, 2H), 4.16–4.13 (m, 1H), 3.97–3.96 (m, 1H), 3.64–3.62 (m, 1H), 3.58–3.56 (m, 1H), 3.54–3.52 (m, 1H), 1.77–1.73 (m, 4H), 1.54–1.51 (m, 4H), 1.38 (s, 9H);MS (M + H^+^) 302.28.

#### Preparation of R'-Xaa-NH-R-NH_2_

The compounds with general structure R'-Xaa-NH-R-NH_2_ were prepared through a first HATU-mediated coupling of intermediates H-Xaa-NH-R-NH-Boc with 2-methyl-3-hydroxybenzoic acid, p-nitrobenzoic acid, benzoic acid or α-naphthoic acid following the general procedure (b). The resulting Boc-protected derivatives were then treated according to the general method for Boc removal (d) to give the target compounds as trifluoroacetate salts. Analytical data and ^1^H-NMR spectra of representative compounds are listed below. NMR spectra of the whole series can be found in the Supplementary Material.

*HMB-Leu-NH-(CH_2_)_2_-NH_2_.* White solid, yield 54%; ^1^H NMR (400 MHz, CDCl_3_) δ 7.30 (dd, *J* = 7.5, 1.4 Hz, 1H), 7.21–7.04 (m, 1H), 6.90 (dd, *J* = 7.5, 1.4 Hz, 1H), 6.55 (s, 1H), 5.19 (s, 3H), 5.02 (s, 1H), 4.41 (t, *J* = 7.6 Hz, 1H), 3.95 (t, *J* = 7.6 Hz, 2H), 3.55 (s, 1H), 3.03 (t, *J* = 7.6 Hz, 2H), 2.31 (s, 3H), 1.98–1.66 (m, 2H), 1.58 (t, *J* = 7.5 Hz, 1H), 1.23–0.86 (m, 6H);MS (M + H^+^) 308.21.

*HMB-Asn-NH-(CH_2_)_2_-NH_2_.* Pale yellow solid, yield 79%; ^1^H NMR (400 MHz, CDCl_3_) δ 7.30 (dd, *J* = 7.5, 1.5 Hz, 1H), 7.22–7.06 (m, 1H), 6.91 (dd, *J* = 7.5, 1.4 Hz, 1H), 6.55 (s, 1H), 5.58 (s, 3H), 5.19 (s, 1H), 5.07 (s, 2H), 4.83–3.91 (m, 2H), 3.61 (s, 1H), 3.14–2.96 (m, 3H), 2.87–2.83 (m, 1H), 2.19 (s, 3H);MS(M + H^+^) 309.17.

*HMB-Phe-NH-(CH_2_)_2_-NH_2_.* White solid, yield 81%; ^1^H NMR (400 MHz, CDCl_3_) δ 7.36–7.33 (m, 1H), 7.30–7.03 (m, 6H), 6.91 (dd, *J* = 7.5, 1.4 Hz, 1H), 6.80 (s, 1H), 5.07 (s, 3H), 4.69–4.67 (m, 1H), 4.30 (s, 1H), 3.97–3.90 (m, 2H), 3.56 (s, 1H), 3.30–3.28 (m, 1H), 3.03–2.78 (m, 3H), 2.31 (s, 3H); MS(M + H^+^) 342.18.

*HMB-Ser-NH-(CH_2_)_2_-NH_2_.* White solid, yield 77%; ^1^H NMR (400 MHz, CDCl_3_)δ 7.18–7.01 (m, 2H), 6.97–6.87 (m, 1H), 5.50 (bs, 1H), 5.21 (s, 3H), 4.99 (s, 1H), 4.75–4.73 (m, 1H), 4.29–4.28 (m, 1H), 4.02–3.75 (m, 3H), 3.54 (s, 1H), 3.05–3.02 (m, 2H), 2.29 (s, 3H); MS (M + H^+^) 282.27.

*HMB-Leu-NH-(CH_2_)_4_-NH_2_.* Pale yellow solid, yield 57%; ^1^H NMR (400 MHz, CDCl_3_)δ 7.30–7.26 (m, 1H), 7.14–7.11 (m, 1H), 6.91–6.89 (m, 1H), 6.03 (s, 3H), 5.26 (s, 1H), 5.23–5.11 (m, 2H), 3.74 (s, 1H), 3.50–3.48 (m, 1H), 3.19–3.17 (m, 1H), 2.82–2.75 (m, 2H), 2.24 (s, 3H), 2.02–1.89 (m, 2H), 1.83–1.58 (m, 4H), 1.54–1.52 (m, 1H), 1.04–0.93 (m, 6H); MS (M + H^+^) 336.31.

*HMB-Asn-NH-(CH_2_)_4_-NH_2_.* White solid, yield 73%; ^1^H NMR (400 MHz, CDCl_3_)δ 7.70 (s, 1H), 7.61 (s, 1H), 7.32–7.28 (m, 1H), 7.15 (t, *J* = 7.5 Hz, 1H), 6.91–6.94 (m, 1H), 5.26 (s, 2H), 4.94 (s, 3H), 4.63–4.58 (m, 1H), 3.31–3.17 (m, 2H), 3.05–3.02 (m, 1H), 2.82–2.65 (m, 3H), 2.32 (s, 3H), 1.86–1.79 (m, 2H), 1.55–1.50 (m, 2H); MS (M + H^+^) 337.32.

*HMB-Phe-NH-(CH_2_)_4_-NH_2_.* White solid, yield 77%; ^1^H NMR (400 MHz, CDCl_3_)δ 7.40–7.36 (m, 1H), 7.34–7.08 (m, 7H), 6.95–6.91 (m, 1H), 5.16 (s, 3H), 4.73–4.70 (m, 1H), 4.39 (s, 1H), 3.41 (s, 1H), 3.28–3.17 (m, 3H), 2.92–2.89 (m, 1H), 2.78–2.75 (m, 2H), 2.31 (s, 3H), 2.02–1.77 (m, 2H), 1.65–1.55 (m, 2H); MS (M + H^+^) 370.22.

*HMB-Ser-NH-(CH_2_)_4_-NH_2_.* Pale yellow solid, yield 44%; ^1^H NMR (400 MHz, CDCl_3_)δ 7.18–7.13 (m, 1H), 7.10–7.05 (m, 1H), 6.88–6.97 (m, 1H), 5.32 (s, 2H), 4.96 (s, 3H), 4.67–4.58 (m, 1H), 4.14–4.10 (m, 1H), 3.90–3.85 (m, 1H), 3.56 (s, 1H), 3.32–3.18 (m, 2H), 2.76–7.68 (m, 2H), 2.32 (s, 3H), 1.99–1.85 (m, 2H), 1.76–1.62 (m, 2H); MS (M + H^+^) 310.17.

*HMB-Leu-NH-cHx-NH_2_.* Pale yellow solid, yield 81%; ^1^H NMR (400 MHz, CDCl_3_)δ 7.40 (s, 1H), 7.30 (dd, *J* = 7.5, 1.5 Hz, 1H), 7.14 (t, *J* = 7.5 Hz, 1H), 6.91 (dd, *J* = 7.5, 1.4 Hz, 1H), 5.70 (s, 3H), 4.96 (s, 1H), 4.72–4.70 (m, 1H), 3.67–3.63 (m, 1H), 3.59–3.45 (m, 1H), 2.36–2.29 (m, 3H), 2.17–2.05 (m, 2H), 1.84–1.55 (m, 7H), 1.07–0.96 (m, 6H); MS (M + H^+^) 362.14.

*HMB-Asn-NH-cHx-NH_2_.* Pale yellow solid, yield 72%; ^1^H NMR (400 MHz, CDCl_3_)δ 7.50 (s, 1H), 7.36 (dd, *J* = 7.5, 1.4 Hz, 1H), 7.17 (t, *J* = 7.5 Hz, 1H), 6.91 (dd, *J* = 7.5, 1.4 Hz, 1H), 5.27 (s, 2H), 5.16 (s, 3H), 4.49–4.45 (m, 1H), 4.32–4.28 (m, 1H), 3.56 (s, 1H), 3.36–3.31 (m, 1H), 3.07–3.03 (m, 1H), 2.68–2.64 (m, 1H), 2.55–2.49 (m, 2H), 2.35–2.22 (m, 5H), 2.03–1.96 (m, 2H), 1.74–1.68 (m, 2H); MS (M + H^+^) 363.32.

*HMB-Phe-NH-cHx-NH_2_.* White solid, yield 68%; ^1^H NMR (400 MHz, CDCl_3_)δ 7.41 (dd, *J* = 7.5, 1.5 Hz, 1H), 7.34–7.24 (m, 4H), 7.24–7.08 (m, 2H), 7.02–6.85 (m, 2H), 5.27 (s, 1H), 5.18 (s, 3H), 4.90 (t, *J* = 3.6 Hz, 1H), 4.19–4.15 (m, 1H), 3.56–3.34 (m, 2H), 3.00–2.97 (m, 1H), 2.40–2.22 (m, 5H), 2.10–2.05 (m, 2H), 1.81–1.76 (m, 2H), 1.48–1.36 (m, 2H); MS (M + H^+^) 396.33.

*HMB-Ser-NH-cHx-NH_2_.* Pale yellow, yield 69%; ^1^H NMR (400 MHz, CDCl_3_)δ 7.32 (dd, *J* = 7.5, 1.4 Hz, 1H), 7.18 (t, *J* = 7.5 Hz, 1H), 6.91 (dd, *J* = 7.5, 1.4 Hz, 1H), 5.04 (s, 1H), 4.75 (s, 1H), 4.68 (s, 3H), 4.31–4.06 (m, 2H), 4.03–3.88 (m, 1H), 3.87–3.66 (m, 1H), 3.61–3.32 (m, 2H), 2.29 (s, 3H), 2.23–2.10 (m, 2H), 2.05–1.79 (m, 4H), 1.67–1.45 (m, 2H); MS (M + H^+^) 336.52.

#### Preparation of R'-Xaa-NH-R-NH-ClNafQ

The final compounds **1**–**48** were obtained by the reaction of intermediates R'-Xaa-NH-R-NH_2_ with 2,3-dichloro-1,4-naphthoquinone following the general procedure (e). All the compounds were purified by RP-HPLC. Analytical data and ^1^H-NMR spectra of representative compounds (**1–16**) are listed below. NMR spectra of the whole series can be found in the Supplementary Material.

*HMB-Leu-NH-(CH_2_)_2_-NH-ClNafQ (***1***).* White solid; yield 58%;mp =111–114 °C; [α]_D_^20^–30.2 (c = 1, MeOH). ^1^H NMR (400 MHz, CDCl_3_)δ 8.29–8.07 (m, 2H), 8.05 (bs, 1H), 7.78–7.66 (m, 2H), 7.23–6.98 (m, 2H), 6.95–6.78 (m, 1H), 5.79 (bs, 1H), 5.71 (bs, 1H), 4.72–4.66 (m, 1H), 3.56–3.45 (m, 4H), 2.29 (s, 3H), 1.85–1.69 (m, 2H), 1.56 (t, *J* = 6.6 Hz, 1H), 0.97–0.87 (m,6H); MS (ESI): [MH]^+^ = 498.12.HPLC (T_r_) 16.76 min.

*NBz-Leu-NH-(CH_2_)_2_-NH-ClNafQ (***2***).* White solid; yield 66%;mp =134–137 °C; [α]_D_^20^–26.4 (c = 1, MeOH); ^1^H NMR (400 MHz, CDCl_3_)δ 8.22–8.13 (m, 4H), 8.08 (d, *J* = 7.4 Hz, 2H), 7.80–7.69 (m, 2H), 7.41 (bs, 1H), 6.80 (bs, 1H), 5.65 (bs, 1H), 3.93–3.86 (m, 1H), 3.89–3.41 (m, 4H), 1.86–1.57 (m, 2H), 1.45–1.43 (m, 1H), 0.97–0.87 (m, 6H); MS (ESI): [MH]^+^ = 513.11; HPLC (T_r_) 18.40 min.

*Bz-Leu-NH-(CH_2_)_2_-NH-ClNafQ (***3***).* White solid; yield 89%;mp =128–130 °C; [α]_D_^20^ –22.4 (c = 1, MeOH); ^1^H NMR (400 MHz, CDCl_3_)δ 8.25–8.10 (m, 2H), 7.89–7.83 (m, 2H), 7.79–7.68 (m, 2H), 7.53–7.358 (m, 3H), 6.54 (s, 1H), 5.45 (s, 1H), 5.31 (s, 1H), 4.23–4.18 (m, 1H), 3.58–3.44 (m, 3H), 3.40–3.36 (m, 1H), 1.68–1.42 (m, 3H), 1.06 (d, *J* = 6.1 Hz, 6H); MS (ESI): [MH]^+^ = 468.03; HPLC (T_r_) 17.71 min.

*1-NaftCO-Leu-NH-(CH_2_)_2_-NH-ClNafQ (***4***).* White solid; yield 74%;mp =145–148 °C; [α]_D_^20^–33.2 (c = 1, MeOH); ^1^H NMR (400 MHz, CDCl_3_)δ 8.52–8.47 (m, 1H), 8.18–8.02 (m, 2H), 7.99–7.93 (m, 2H), 7.82–7.78 (m, 1H), 7.74–7.64 (m, 2H), 7.58–7.46 (m, 3H), 7.21 (s, 1H), 7.05 (s, 1H), 5.75 (s, 1H), 4.52–4.47 (m, 1H), 3.64–3.39 (m, 4H), 1.99–1.86 (m, 1H), 1.73–1.34 (m, 2H), 1.12–0.93 (m, 6H); MS (ESI): [MH]^+^ = 518.15; HPLC (T_r_) 19.11 min.

*HMB-Asn-NH-(CH_2_)_2_-NH-ClNafQ (***5***).* White solid; yield 66%;mp =128–130 °C; [α]_D_^20^–42.1 (c = 1, MeOH); ^1^H NMR (400 MHz, CDCl_3_)δ 8.57 (s, 1H), 8.34–8.23 (m, 1H), 7.95–7.86 (m, 1H), 7.67–7.58 (m, 2H), 7.32–7.29 (m, 1H), 7.14–7.10 (m, 1H), 6.89–6.81 (m, 1H), 5.50 (s, 1H), 5.35 (s, 1H), 5.23–5.18 (m, 1H), 4.73 (s, 2H), 3.62–3.54 (m, 3H), 3.46–3.40 (m, 1H), 3.03–2.98 (m, 1H), 2.51–2.46 (m, 1H), 2.33 (s, 3H); MS(ESI): [MH]^+^ = 499.24; HPLC (T_r_) 13.21 min.

*NBz-Asn-NH-(CH_2_)_2_-NH-ClNafQ (***6***).* White solid; yield 52%;mp =168–170 °C; [α]_D_^20^–38.8 (c = 1, MeOH); ^1^H NMR (400 MHz, CDCl_3_)δ 8.74 (s, 1H), 8.20–8.05 (m, 6H), 7.79–7.68 (m, 2H), 5.69–5.66 (m, 3H), 5.30 (s, 1H), 4.72–4.66 (m, 1H), 3.70–3.56 (m, 1H), 3.55–3.32 (m, 3H), 2.96–2.91 (m, 1H), 2.50–2.45 (m, 1H); MS (ESI): [MH]^+^ = 514.08; HPLC (T_r_) 13.66 min.

*Bz-Asn-NH-(CH_2_)_2_-NH-ClNafQ (***7***).* White solid; yield 48%;mp =165–167 °C; [α]_D_^20^ –26.6 (c = 1, MeOH); ^1^H NMR (400 MHz, CDCl_3_)δ 8.27–8.08 (m, 2H), 7.87 (dd, *J* = 7.4, 1.3 Hz, 2H), 7.74–7.65 (m, 2H), 7.41–7.21 (m, 3H), 7.24 (s, 1H), 6.47 (s, 1H), 6.00 (s, 1H), 5.02 (s, 2H), 4.87–4.82 (m, 1H), 3.60–3.37 (m, 4H), 3.13–3.08 (m, 1H), 2.86–7.82 (m, 1H);MS (ESI): [MH]^+^ = 469.12; HPLC (T_r_) 14.30 min.

*1-NaftCO-Asn-NH-(CH_2_)_2_-NH-ClNafQ (***8***).* White solid; yield 87%;mp =178–180 °C; [α]_D_^20^–42.8 (c = 1, MeOH); ^1^H NMR (400 MHz, CDCl_3_) δ 8.57–8.41 (m, 2H), 8.20–8.04 (m, 2H), 8.00–7.87 (m, 2H), 7.84–7.80 (m, 1H), 7.76–7.60 (m, 2H), 7.61–7.44 (m, 3H), 5.66 (s, 1H), 5.38 (s, 2H), 4.59–4.45 (m, 1H), 4.17 (s, 1H), 3.60–3.51 (m, 1H), 3.45–3.34 (m, 4H), 2.91–2.89 (m, 1H);MS (ESI): [MH]^+^ = 519.14; HPLC (T_r_) 15.43 min.

*HMB-Phe-NH-(CH_2_)_2_-NH-ClNafQ (***9***).* White solid; yield 72%;mp =121–124 °C; [α]_D_^20^–31.4 (c = 1, MeOH); ^1^H NMR (400 MHz, CDCl_3_) δ 8.17–8.02 (m, 2H), 7.72–7.66 (m, 2H), 7.35–7.04 (m, 8H), 6.90 (dd, *J* = 7.5, 1.4 Hz, 1H), 6.20 (s, 1H), 5.05 (s, 1H), 4.77–4.68 (m, 1H), 3.64–3.40 (m, 4H), 3.42–3.22 (m, 2H), 2.95–2.92 (m, 1H), 2.28 (s, 3H); MS (ESI): [MH]^+^ = 532.02; HPLC (T_r_) 16.91 min.

*NBz-Phe-NH-(CH_2_)_2_-NH-ClNafQ (***10***).* White solid; yield 59%;mp =141–144 °C; [α]_D_^20^–28.9 (c = 1, MeOH); ^1^H NMR (400 MHz, CDCl_3_)δ 8.83 (s, 1H), 8.25–8.15 (m, 3H), 8.09 (dd, *J* = 7.5, 1.6 Hz, 1H), 8.03 (d, *J* = 7.5 Hz, 2H), 7.84–7.80 (m, 1H), 7.70–7.66 (m, 1H), 7.23–7.20 (m, 2H), 7.16–7.04 (m, 3H), 6.23 (s, 1H), 4.69 (s, 1H), 4.03–3.98 (m, 1H), 3.68–3.50 (m, 2H), 3.50–3.42 (m, 1H), 3.45–3.32 (m, 1H), 3.25–3.15 (m, 1H), 2.98–2.96 (m, 1H); MS (ESI): [MH]^+^ = 547.30; HPLC (T_r_) 18.73 min.

*Bz-Phe-NH-(CH_2_)_2_-NH-ClNafQ (***11***).* White solid; yield 86%;mp =122–124 °C; [α]_D_^20^–23.4 (c = 1, MeOH); ^1^H NMR (400 MHz, CDCl_3_)δ 8.23–8.13 (m, 1H), 8.05–7.83 (m, 3H), 7.77–7.65 (m, 3H), 7.52–7.48 (m, 3H), 7.31–7.19 (m, 2H), 7.19–7.11 (m, 3H), 4.87 (s, 1H), 4.55 (s, 1H), 4.10–4.02 (m, 1H), 3.57–3.41 (m, 4H), 3.30–3.27 (m, 1H), 2.98–2.96 (m, 1H);MS (ESI): [MH]^+^ = 502.15; HPLC (T_r_) 18.05 min.

*1-NaftCO-Phe-NH-(CH_2_)_2_-NH-ClNafQ (***12***).* White solid; yield 66%;mp =130–134 °C; [α]_D_^20^–33.2 (c = 1, MeOH); ^1^H NMR (400 MHz, CDCl_3_)δ 8.51 (dd, *J* = 7.4, 1.5 Hz, 1H), 8.39 (s, 1H), 8.23–8.03 (m, 2H), 7.95–7.94 (m, 1H), 7.84–7.81 (m, 1H), 7.74–7.60 (m, 2H), 7.58–7.56 (m, 2H), 7.49–7.48 (m, 1H), 7.28–7.18 (m, 3H), 7.10–7.01 (m, 3H), 6.67 (s, 1H), 5.56 (s, 1H), 4.81–4.76 (m, 1H), 3.59–3.51 (m, 1H), 3.51–3.39 (m, 3H), 3.33–3.30 (m, 1H), 3.03–3.00 (m, 1H); MS (ESI): [MH]^+^ = 552.16; HPLC (T_r_) 19.65 min.

*HMB-Ser-NH-(CH_2_)_2_-NH-ClNafQ (***13***).* White solid; yield 76%;mp =117–120 °C; [α]_D_^20^–45.1 (c = 1, MeOH); ^1^H NMR (400 MHz, CDCl_3_)δ 8.11–7.99 (m, 2H), 7.70–7.64 (m, 2H), 7.36–7.32 (m, 1H), 7.10–7.07 (m, 2H), 6.91–6.89 (m, 1H), 6.08 (s, 1H), 5.06 (s, 1H), 4.28–4.17 (m, 1H), 4.19–4.17 (m, 1H), 3.78–3.76 (m, 1H), 3.56–3.40 (m, 4H), 2.32 (s, 3H); MS (ESI): [MH]^+^ = 472.08; HPLC (T_r_) 12.84 min.

*NBz-Ser-NH-(CH_2_)_2_-NH-ClNafQ (***14***).* White solid; yield 56%;mp =142–146 °C; [α]_D_^20^–39.8 (c = 1, MeOH); ^1^H NMR (400 MHz, CDCl_3_)δ 8.21–8.10 (m, 3H), 8.08–8.00 (m, 3H), 7.91 (s, 1H), 7.79–7.62 (m, 2H), 5.56 (s, 1H), 5.21 (s, 1H), 4.26–4.08 (m, 2H), 3.99–3.96 (m, 1H), 3.68–3.50 (m, 2H), 3.50–3.41 (m, 1H), 3.41–3.27 (m, 1H);MS (ESI): [MH]^+^ = 487.09; HPLC (T_r_) 13.22 min.

*Bz-Ser-NH-(CH_2_)_2_-NH-ClNafQ (***15***).* White solid; yield 69%;mp =155–157 °C; [α]_D_^20^ –30.6 (c = 1, MeOH); ^1^H NMR (400 MHz, CDCl_3_)δ 8.25–8.19 (m, 1H), 8.10–8.05 (m, 1H), 7.74–7.70 (m, 4H), 7.66–7.64 (m, 1H), 7.48–7.41 (m, 1H), 7.36 (t, *J* = 7.4 Hz, 2H), 5.71 (s, 1H), 5.54 (s, 1H), 4.74–4.65 (m, 1H), 4.24–4.20 (m, 1H), 3.84–3.80 (m, 1H), 3.62–3.32 (m, 4H); MS (ESI): [MH]^+^ = 442.11; HPLC (T_r_) 13.97 min.

*1-NaftCO-Ser-NH-(CH_2_)_2_-NH-ClNafQ (***16***).* White solid; yield 65%;mp =163–166 °C; [α]_D_^20^–41.5 (c = 1, MeOH);^1^H NMR (400 MHz, CDCl_3_) δ 8.78 (dd, *J* = 7.4, 1.5 Hz, 1H), 8.15–7.99 (m, 2H), 7.95–7.94 (m, 1H), 7.79–7.77 (m, 1H), 7.72–7.62 (m, 4H), 7.56–7.41 (m, 3H), 5.68 (s, 1H), 5.08–5.05 (m, 2H), 4.20–4.17 (m, 1H), 3.96–3.94 (m, 1H), 3.67–3.61 (m, 1H), 3.49–3.47 (m, 3H); MS (ESI): [MH]^+^ =  492.12; HPLC (T_r_) 14.86 min.

### Biological assay

#### Proteasome purification and subunit inhibition

Proteasomes were isolated and purified from lymphoblastoid cell lines (LCL) as previously described^31^. Suc-LLVY-AMC, Boc-LRR-AMC and Z-LLE-AMC (Sigma-Aldrich, Milano, Italy) were used to determine chymotrypsin-like, trypsin-like and post-acidic proteasome activities, respectively. Substrates were incubated at 37 °C for 30 min with proteasomes, untreated or pretreated with 0.1–100 µm of test compounds **1**–**48** and reference inhibitor MG132, in activity buffer. Fluorescence was determined by a fluorimeter (Spectrafluor plus, Tecan, Salzburg, Austria), using an excitation of 360 nm and emission of 465 nm. Activity was evaluated in fluorescence units and the inhibitory capacity of the compounds is expressed as IC_50_.

#### Growth inhibition assays

Cell growth inhibition assays were carried out using the breast cancer cell line MDA and ovarian cancer cell line A2780. Cells were obtained from ATCC (Manassas, VA) and maintained in DMEM and RPMI respectively, supplemented with 10% foetal bovine serum, penicillin (100 U ml^−1^), streptomycin (100 U ml^−1^) and glutamine (2 mm); incubation was performed at 37 °C in a 5% CO_2_ atmosphere. Cells were routinely passaged every 3 d at 70% confluence; 0.05% trypsin-EDTA was used. The antiproliferative activity of new molecules was tested with the 3-(4,5-dimethylthiazol-2-yl)-2,5-diphenyltetrazolium bromide (MTT) assay. Cells were seeded in triplicate in 96-well plates at a density of 15 × 10^3^ in 50 µl of complete medium. Stock solutions (10 mm) of selected analogues were made in DMSO and diluted in complete medium to give final concentrations of 10 and 100 µm. MG-132 was employed as a control. Untreated cells were placed in every plate as a negative control. The cells were exposed to the compounds, in 100 µl total volume, for 72 h, and then 25 µl of a 12 mm solution of MTT was added. After 2 h of incubation, 100 µl of lysing buffer (50% DMF +20% SDS, pH 4.7) was added to convert the MTT solution into a violet-coloured formazan. After an additional 18 h the solution absorbance, proportional to the number of live cells, was measured by spectrophotometer at 570 nm and converted into % of growth inhibition.

### Docking

The equilibrium geometry of selected molecules was obtained using semi-empirical PM3 calculations; the molecules were subsequently docked to both β1 and β5 binding sites of 20 S proteasome. The simulation was performed utilising the crystal structures of PDB codes **1G65**^32^ and **3E47**^33^ for β1 and β5 binding sites, respectively. All molecules were placed in β1 and β5 binding sites using a pharmacophore query derived from the bound inhibitors epoxomicin (structure 1G65) and homobelactosin C (structure 3E47), respectively, as a filter for docking placement. Epoxomicin and homobelactosin C inhibitors have been chosen because they share size/shape similarities with the present molecules. Before the simulation, hydrogen atoms were added to the inner part of the enzyme and the energy of the structure was minimised keeping fixed the atoms of the main frame and using the MMFF94 molecular mechanics force field^34^. Out of 50 unique poses obtained, the 10 having the highest score on the base of the value assumed by the enthalpic contribution to the free energy of binding were retained. These poses were in turn rescored considering the estimation of the free energy of binding of the ligand, i.e. the sum of the electrostatic and dispersive interaction energy between the ligand and the target as well as the intramolecular energy of the ligand due to changes in its conformation. All the calculations were performed using MOE-Dock integrated into the MOE system of programs [MOE, Chemical Computing Group, release 2016.08].

## Results and discussion

The synthesised compounds were evaluated for their potency in inhibiting the β1, β2 and β5 catalytic activities of the 20 S proteasome isolated from LCLs^31^. Specific fluorogenic substrates for each active site were employed: Suc-LLVY-AMC (for the ChT-L), Boc-LRR-AMC (for the T-L) and Z-LLE-AMC (for the PGPH). The proteasome was pretreated with increasing concentrations (0.1–100 µm) of the new naphthoquinone amino acid derivatives and MG132 as a reference inhibitor in an activity buffer. Substrates were then incubated with the proteasome at 37 °C for 30–180 min. Substrate degradation was evaluated in fluorescence units. The inhibitory activity of all compounds is expressed here as IC_50_. From the data reported in Table 1, after 30 min of incubation, it can be observed that some of the naphthoquinone analogues present a significant biological response. The inhibition of the PGPH and ChT-L activities is remarkable for some of the derivatives. In particular, the inhibition of the β1 and β5 subunits is interesting for compounds **4**, **6**, **9** and **10** with IC_50_ values lower than 1 µm. Generally, the molecules bearing the ethylenediamine linker (**1–16**) displayed a quite good activity against post-acidic and chymotryptic sites of the proteasome and retain a detectable (IC_50_< 100 µm) inhibition of the tryptic-like activity in the β2 subunit. The most effective β1 inhibitors of the subseries with the ethylenediamine spacer present leucine or phenylalanine, while for the β5 inhibition are favourable asparagine and phenylalanine.

Regarding the Nα substituents, the NBz group is slightly preferred as indicated by the analogue **10** which is the most potent inhibitor of the whole series. A similar structure-activity relationship profile is evident within the naphthoquinone amino acid derivatives bearing the butylendiaminic linker (**17–32**). Also in this subset, the inhibition is mainly directed to the catalytic cavity β1 and β5, while the answer against the tryptic activity is irrelevant. Generally, it can be speculated that the increase of the distance between the pharmacophoric unit and the aminoacidic residue at the P1 position is negative for the interaction with the catalytic pocket of the enzyme complex. Finally, analogues **33**–**48** are the least active of the whole series with only a mild inhibition of the β5 subsite for derivatives with leucine (i.e. **34**–**36**) and of the post-acidic activity (β1) for products having serine as the central residue (i.e. **46**–**48**). Clearly, the length and molecular rigidity of the cyclohexyldiamine bridge are not favourable for the interaction with the catalytic proteasome pockets.

**Figure 2.**
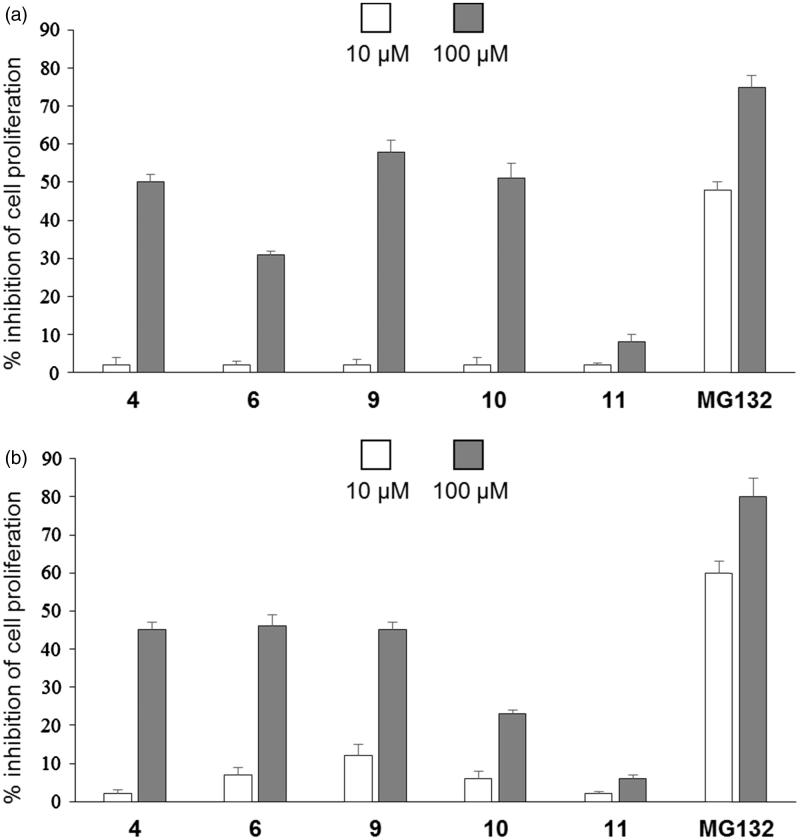
Effect of compounds **4**, **6**, **9**–**11** on cell proliferation. (a) MDA and (b) A2780 tumour cells cultured for 3 d in the presence or absence of the indicated concentrations of compounds. The means of three independent experiments performed in duplicate are shown.

Considering the fundamental role of the proteasome for cell viability and proliferation, we investigated the antiproliferative activities of five selected compounds (**4**, **6** and **9**–**11**) on MDA and A2780 tumour cells compared to the reference aldehydic inhibitor MG132^35^. The choice of analogues has been carried out in view of their ability to inhibit the chymotryptic subunit of the proteasome, directly related to the anti-cancer activity. The cells were treated with 10 or 100 µm concentrations of the selected naphthoquinone derivatives and MG132. After 3 d, cell proliferation was evaluated and, as shown in Figure 2(a and b), compounds **4**, **6** and **9** were shown to inhibit cell proliferation at levels slightly lower than the reference pseudotripeptide MG132 in both tumoural cell lines at 100 µm. No significant antiproliferative activity was observed at 10 µm.

A general analysis of the structure-activity relationship of this new series of amino acidic derivatives suggests that the naphthoquinone pharmacophoric unit can function as potential electrophilic substrate for the proteasome N-terminal catalytic threonine. The strength of the interaction with the enzyme subsites depends on the molecular components that bind the naphthoquinonic group. The ethylenediamine binder between the amino acid residue and the pharmacophore was shown to be the most effective since increasing the alkyl chain with the insertion of the butylenediamine and making it more rigid with the cyclohexyldiamine, a progressive reduction of activity was observed. The biological response is more evident for phenylalanyl and asparagyl derivatives that display a significant preference for the chymotryptic and post-acidic proteasome subunits. In addition, the different Nα-aryl groups, with variable physicochemical characteristics, poorly influence the capacity of inhibition of the new compounds. It can reasonably be assumed that the inhibition is reversible by analysing the data obtained with the docking experiments that exclude a covalent bond between the inhibitor and the enzyme (see below). In addition, the enzymatic inhibition assays of catalytic subsites of the isolated enzyme confirm that the IC_50_ values for the naphthoquinone amino acid derivatives, progressively decrease during the time of incubation.

**Figure 3.**
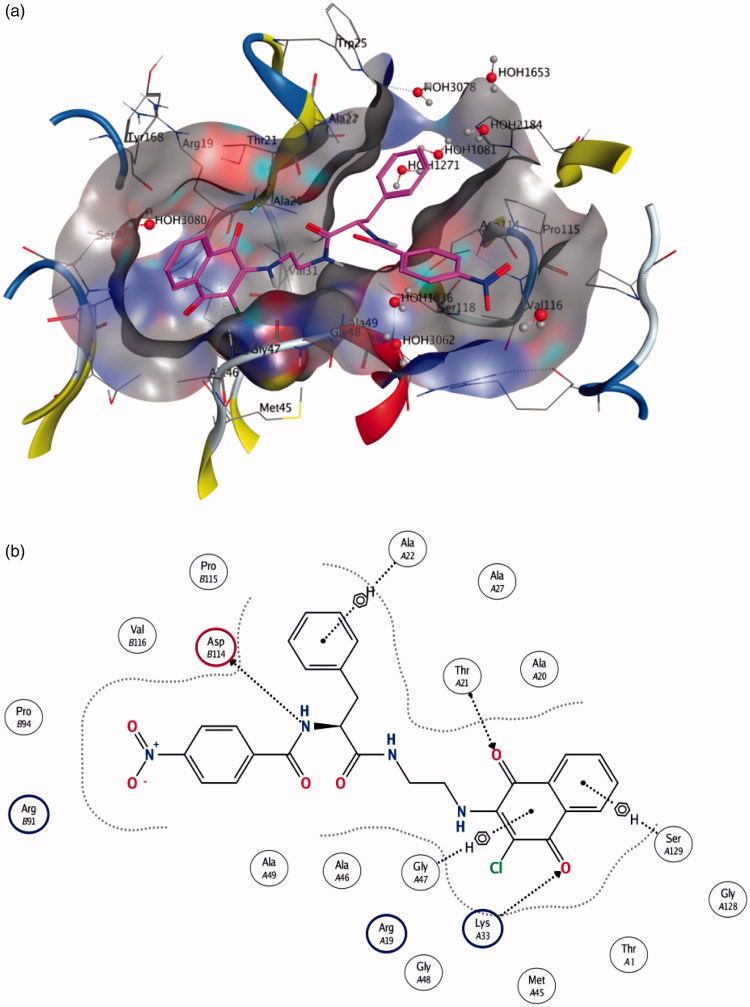
(a) Molecule **10** in the β1 active site (best pose). (b) Schematic view of the interactions between the receptor and the docked molecule.

A computational docking study was performed for selected compounds (**10**, **16**, **26** and **42**) on the basis of their different molecular characteristics and biological profile. The results of the docking study of the most active molecule **10** to both β1 and β5 binding sites of 20 S proteasome seem to support the previous considerations. In the docking simulation to the β1 site, five poses (out of 10) having the highest docking scores are characterised by the presence of the naphthoquinonic moiety located ca. 3.5 Å away from the nucleophilic threonine residue. In view of the fact that the protein frame is kept rigid during the simulation, such a distance is compatible with an H-bonding interaction. Molecule **10** docked to the enzyme and a scheme of the ligand-enzyme interactions are depicted in Figure 3(a and b), respectively. The ligand **10**-binding pocket presents a Y shape, that is appropriate to well fit the molecule conformation. Moreover, it is formed by a number of amino acid residues, i.e. Thr21, Ala20, Gli47, Lys33, Asp114, which are found to be involved in epoxomicin-enzyme interactions as well. Additionally, the aromatic rings seem to play an important role in “anchoring” the molecule inside the binding pocket through C-H … π short contacts (Figure 3(a)).

**Figure 4.**
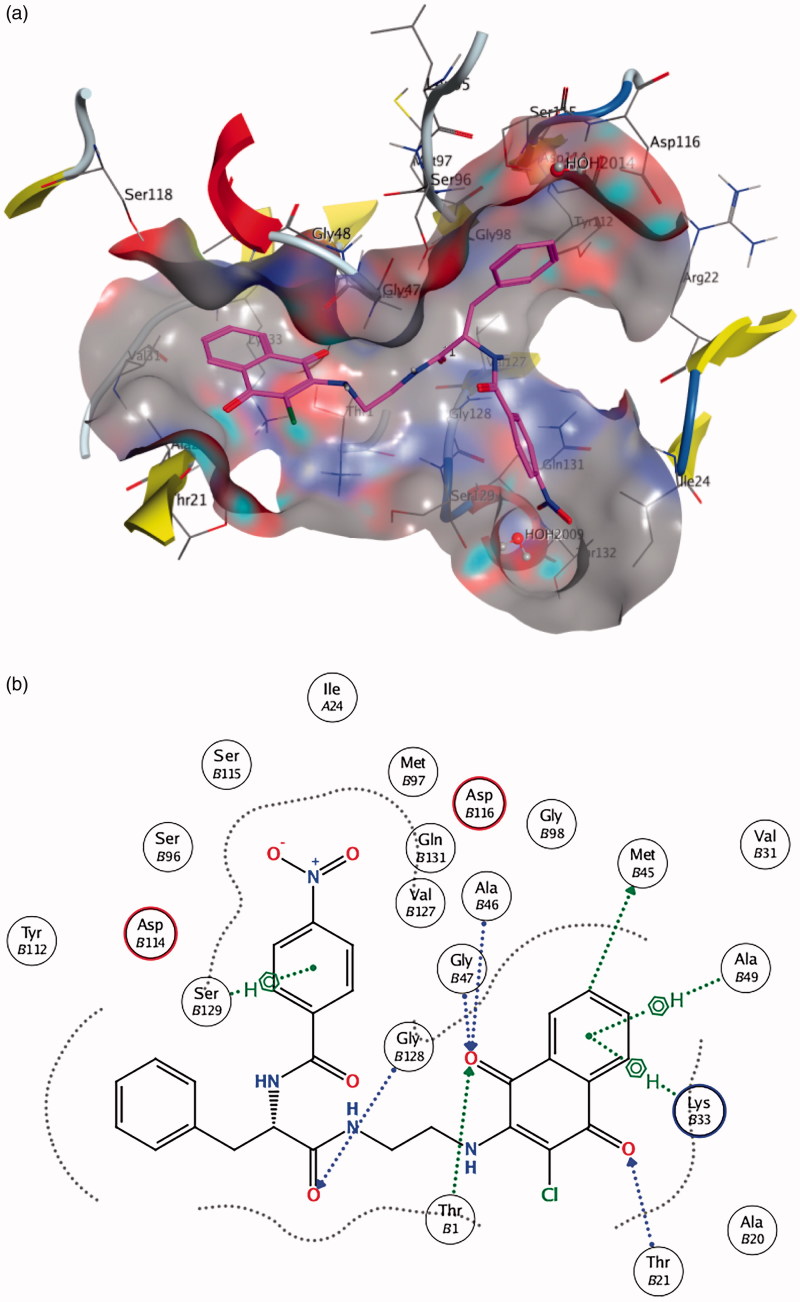
(a) Molecule **10** in the β5 active site. (b) Schematic view of the interactions between the receptor and the docked molecule.

The same considerations can be drawn from the molecule **10** docked to the β5 site. Indeed, the interactions with the binding-site residues (Figure 4) are even more significant: in this case, for instance, a direct tight interaction with the active threonine has been found, with a ligand-residue distance of some 2.8 Å. A number of additional short contacts can be observed between the functional groups of the ligand and the amino acids constituting the homobelactosin receptor, e.g. Ser129, Gly47, Lys33 and, just like the β1 case, C-H … π interactions involving the ligand aromatic parts are established (Figure 4(b)).

**Figure 5.**
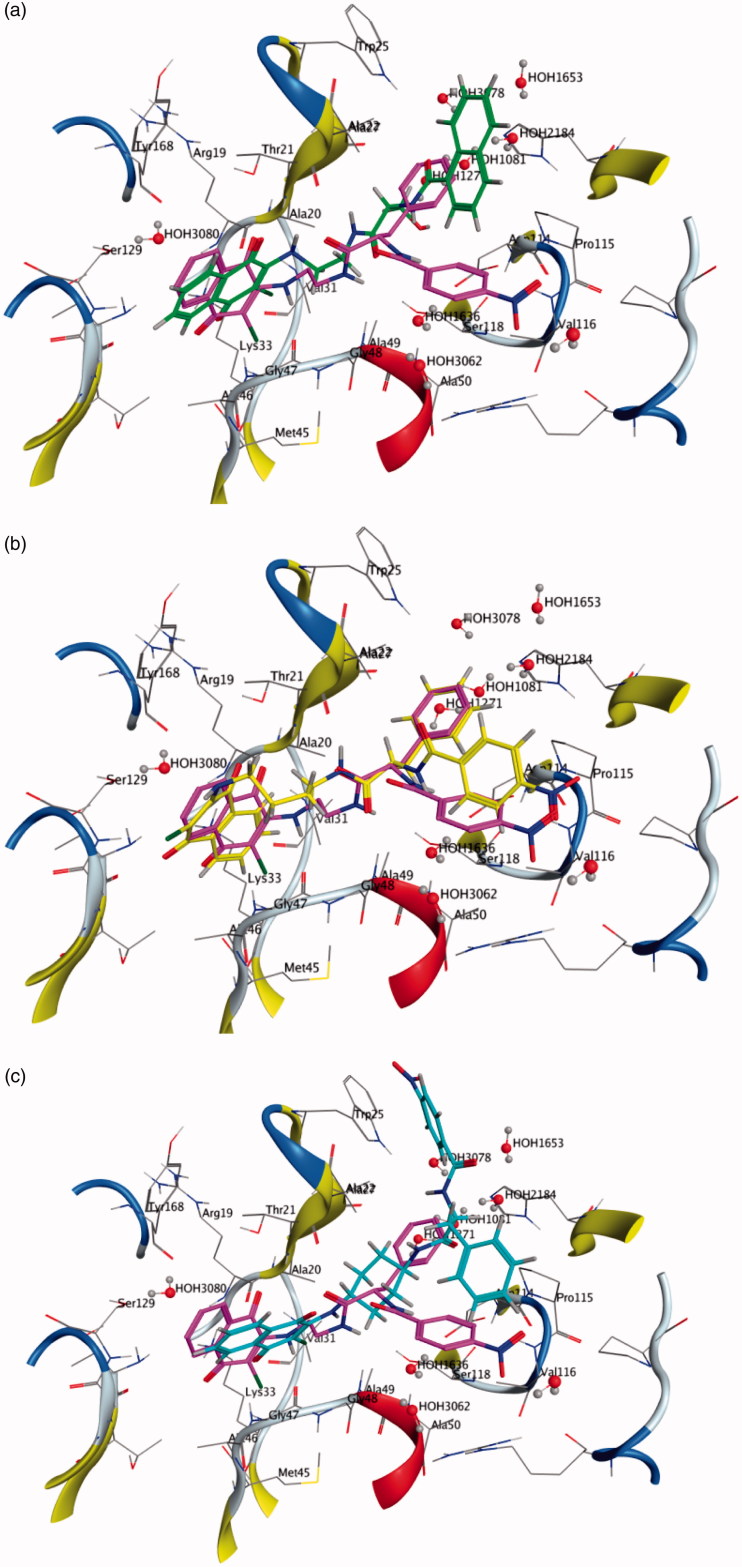
Molecule **16** (a), **26** (b) and **42** (c) docked into the ?1 binding site. Molecule **10** docked in the same binding site is reported for comparison.

**Figure 6.**
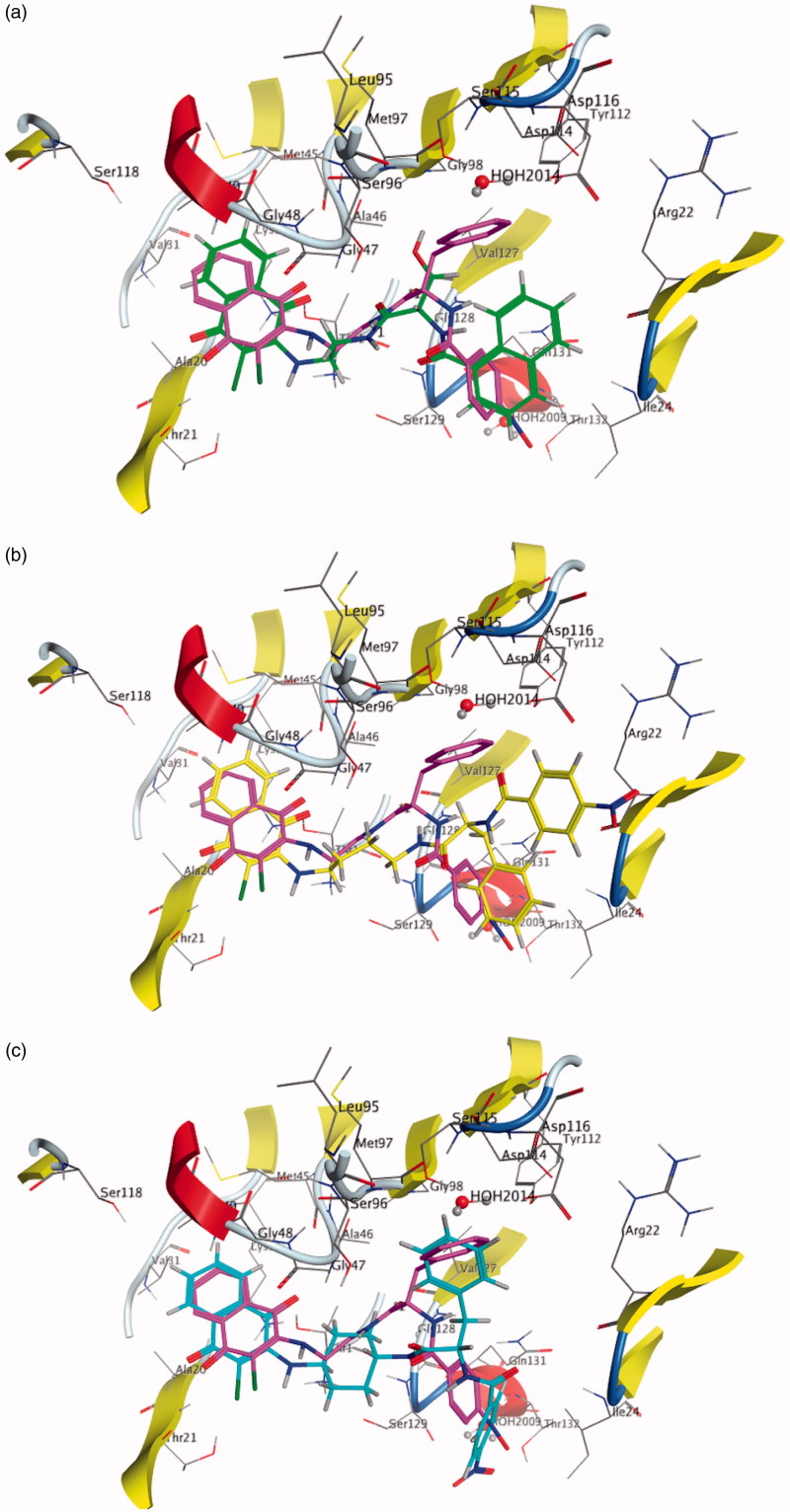
Molecule **16** (a), **26** (b) and **42** (c) docked into the β5 binding site. Molecule **10** docked in the same binding site is reported for comparison.

The different biological behaviour of molecules **16**, **26** and **42** can be rationalised by directly comparing their best-docked poses with **10** (Figures 5 and 6). The inactive molecule **16** does not fit in both the β1 and the β5 sites, the terminal naphthyl group being located in an area outside the binding pocket (Figures 5(a) and 6(a)). As for molecules **26** and **42**, they share with **10** the terminal parts (ClNafQ and NBz-Phe) but with linkers different in length (molecule **26**) or in flexibility (molecule **42**). As a consequence, **26** can achieve a not very strained conformation to accommodate itself into the receptor in a position close to **10,** due to the flexibility of the alkyl chain (Figures 5(b) and 6(b)); conversely, in **42** the presence of a rigid cyclohexane linker restricts the conformational adjustments adoptable by the molecule.

## Conclusion

In summary, this work reports the design, synthesis and molecular modelling study of 48 amino acid derivatives assayed as inhibitors of three major catalytic activities of the proteasome. These compounds have at the Cα position of the aminoacidic residues a 2-chloronaftoquinone group pharmacophoric unit primary responsible for the interaction with the active subunits of the enzymatic complex. The same scaffold is present in non-peptidic molecules and pseudodipeptide derivatives previously reported as inhibitors of the 20 S proteasome. Data on the biological response of some analogues of this new series showed an interesting inhibition of the proteasome. Derivatives **4**, **6**, **9** and **10** were the most active against the β1 and β5 subunits, with a biological profile that makes them potentially capable of operating such as antineoplastic agents. The molecular structure of the new inhibitors allows further studies on the structure-activity relationships.

## Supplementary Material

IENZ_1334649_Supplementary_Information.pdf
