# Supplementary material for: Naphthoquinone amino acid derivatives, synthesis and biological activity as proteasome inhibitors
Source: J Enzyme Inhib Med Chem. 2017 Jun 28;32(1):865–77. doi: 10.1080/14756366.2017.1334649 (PMC6445160; doi:10.1080/14756366.2017.1334649)
Supplement: IENZ_1334649_Supplementary_Information.pdf [file IENZ_A_1334649_SM3941.pdf]

SUPPLEMENTARY DATA FOR

Naphthoquinone amino acid derivatives, synthesis and biological activity as proteasome inhibitors

Mauro Marastoni,[#] Claudio Trapella,[#] Alessandra Scotti,[#] Anna Fantinati,[#] Valeria Ferretti,[#] Erika Marzola,[#] Eleonora Gallerani,[§] Riccardo Gavioli,[§] and Delia Preti[*#]

[#]Department of Chemical and Pharmaceutical Sciences, Via Mortara 17, 44121 Ferrara, Italy.
[§]Department of Life Sciences and Biotechnology, Via Luigi Borsari 46, 44121 Ferrara, Italy.

CONTENTS

**Analytical data of R'-Xaa-NH-R-NH$_2$**	2S
**Analytical data of the final compounds with general formula R'-Xaa-NH-R-NH-ClNafQ (17-48)**	5S

Analytical data of R'-Xaa-NH-R-NH$_2$

NBz-Leu-NH-(CH$_2$)$_2$-NH$_2$: white solid, yield 62%; ^{1}H NMR (400 MHz, CDCl$_3$) δ 8.32 (d, *J* = 7.4 Hz, 2H), 8.12 (d, *J* = 7.4 Hz, 2H), 6.13 (s, 3H), 5.72 (t, *J* = 7.2 Hz, 1H), 5.45 (s, 1H), 5.28 (s, 1H), 3.96 (t, *J* = 4.6 Hz, 1H), 3.89 (t, *J* = 4.6 Hz, 1H), 3.07 (t, *J* = 4.6 Hz, 2H), 1.78 (t, *J* = 7.3 Hz, 1H), 1.65 (t, *J* = 7.3 Hz, 1H), 1.55 – 1.36 (m, 1H), 1.01 (d, *J* = 6.3 Hz, 6H);MS (M+H$^+$) 323.30.

Bz-Leu-NH-(CH$_2$)$_2$-NH$_2$: white solid, yield 74%; ^{1}H NMR (400 MHz, CDCl$_3$) δ 7.94–7.73 (m, 2H), 7.55–7.26 (m, 3H), 6.13 (s, 3H), 5.45-5.51 (m, 2H), 5.22 (s, 1H), 3.94 (t, *J* = 4.7 Hz, 1H), 3.89 (t, *J* = 4.7 Hz, 1H), 3.06 (t, *J* = 4.7 Hz, 2H), 1.86 – 1.42 (m, 3H), 1.17 – 0.87 (m, 6H); MS (M+H$^+$) 278.18.

1-NaftCO-Leu-NH-(CH$_2$)$_2$-NH$_2$: pale yellow solid, yield 54%; ^{1}H NMR (400 MHz, CDCl$_3$) δ 9.04–8.82 (m, 1H), 7.98-7.96 (m, 1H), 7.89–7.74 (m, 2H), 7.66–7.27 (m, 3H), 6.23 (s, 3H), 5.55 (t, *J* = 6.2 Hz, 1H), 5.45 (s, 1H), 5.26 (s, 1H), 3.94 (t, *J* = 4.7 Hz, 1H), 3.90 (t, *J* = 4.7 Hz, 1H), 3.07 (t, *J* = 4.7 Hz, 2H), 1.94–1.67 (m, 2H), 1.63-1.60 (m, 1H), 1.17–0.83 (m, 6H); MS (M+H$^+$) 328.27.

NBz-Asn-NH-(CH$_2$)$_2$-NH$_2$: white solid, yield 62 %; ^{1}H NMR (400 MHz, CDCl$_3$) δ 8.33 (d, *J* = 7.4 Hz, 2H), 8.20 (d, *J* = 7.4 Hz, 2H), 7.28 (s, 1H), 5.71 (s, 2H), 5.11 (s, 3H), 4.68 (t, *J* = 3.2 Hz, 1H), 3.87 (t, *J* = 4.8 Hz, 1H), 3.79 (t, *J* = 4.8 Hz, 1H), 3.70 (s, 1H), 3.25-3.02 (m, 3H), 2.82-2.79 (m, 1H); MS(M+H$^+$) 324.27.

Bz-Asn-NH-(CH$_2$)$_2$-NH$_2$: white solid, yield 59 %; ^{1}H NMR (400 MHz, CDCl$_3$) δ 7.96–7.76 (m, 2H), 7.65–7.28 (m, 3H), 5.99–5.90 (m, 4H), 5.59 (s, 1H), 5.38 (s, 2H), 5.33 (s, 1H), 3.99 (t, *J* = 4.4 Hz, 1H), 3.90 (t, *J* = 4.4 Hz, 1H), 3.09-3.05 (m, 3H), 2.85-2.83 (m, 1H); MS(M+H$^+$) 279.17.

1-NaftCO-Asn-NH-(CH$_2$)$_2$-NH$_2$: white solid, yield 72 %; ^{1}H NMR (400 MHz, CDCl$_3$) δ 8.84–8.78 (m, 1H), 8.07 (dd, *J* = 7.5, 1.6 Hz, 1H), 7.96-7.94 (m, 1H), 7.88–7.74 (m, 1H), 7.62–7.35 (m, 3H), 6.06–5.88 (m, 4H), 5.58 (s, 1H), 5.41 (s, 2H), 5.38 (s, 1H), 3.98-3.97 (m, 2H), 3.27–2.97 (m, 3H), 2.87-2.85 (m, 1H); MS(M+H$^+$) 329.21.

NBz-Phe-NH-(CH$_2$)$_2$-NH$_2$: pale yellow solid, yield 46 %; ^{1}H NMR (400 MHz, CDCl$_3$)δ 8.35 (d, *J* = 7.4 Hz, 2H), 8.22 (d, *J* = 7.4 Hz, 2H), 7.39–7.21 (m, 4H), 7.22–7.09 (m, 1H), 6.49 (s, 1H), 4.90-4.87 (m, 1H), 4.09–3.94 (m, 4H), 3.91 (t, *J* = 5.4 Hz, 1H), 3.34-3.32 (m, 1H), 3.10-2.87 (m, 3H); MS(M+H$^+$) 357.12.

Bz-Phe-NH-(CH$_2$)$_2$-NH$_2$: white solid, yield 67 %; ^{1}H NMR (400 MHz, CDCl$_3$)δ 7.97–7.79 (m, 2H), 7.46-7.42 (m, 3H), 7.34–7.10 (m, 6H), 5.21 (bs, 3H), 4.72-4.70 (m, 1H), 4.16 (s, 1H), 3.88-3.85 (m, 2H), 3.26-3.24 (m, 1H), 3.19–3.04 (m, 2H), 2.89 (m, 1H); MS (M+H$^+$) 312.23.

1-NaftCO-Phe-NH-(CH$_2$)$_2$-NH$_2$: white solid, yield 57 %; ^{1}H NMR (400 MHz, CDCl$_3$)δ 8.89–8.85 (m, 1H), 8.03-8.01 (m, 1H), 7.92-7.90 (m, 1H), 7.81-7.79 (m, 1H), 7.69–7.65 (m, 3H), 7.24-7.22 (m, 2H), 7.19–6.95 (m, 3H), 5.00-4.98 (m, 1H), 4.61 (s, 3H), 3.98-3.97 (m, 1H), 3.91-3.88 (m, 2H), 3.01-2.99 (m, 2H), 2.47-2.46 (m, 1H); MS (M+H$^+$) 362.12.

NBz-Ser-NH-(CH$_2$)$_2$-NH$_2$: white solid, yield 84 %; ^{1}H NMR (400 MHz, CDCl$_3$)δ 8.32 (d, *J* = 7.4 Hz, 2H), 8.15 (d, *J* = 7.4 Hz, 2H), 7.05 (s, 1H), 6.25 (s, 3H), 5.09 (s, 1H), 4.44-4.42 (m, 1H), 4.29-4.27 (m, 1H), 3.94-3.92 (m, 2H), 3.86-3.84 (m, 1H), 3.02-3.00 (m, 2H); MS (M+H$^+$) 297.12.

Bz-Ser-NH-(CH_2)$_2$-NH_2: white solid, yield 66 %; ^{1}H NMR (400 MHz, $CDCl_3$)δ 8.01–7.86 (m, 2H), 7.56–7.39 (m, 3H), 6.08 (s, 3H), 5.39-5.20 (m, 2H), 5.15 (s, 1H), 4.16-4.13 (m, 1H), 4.06-4.02 (m, 1H), 4.00–3.85 (m, 2H), 3.06-3.02 (m, 2H); MS (M+H$^+$) 252.17.

1-NaftCO-Ser-NH-(CH_2)$_2$-NH_2: white solid, yield 49 %; ^{1}H NMR (400 MHz, $CDCl_3$)δ 9.13-9.10 (m, 1H), 7.96-7.94 (m, 1H), 7.87–7.74 (m, 2H), 7.69-7.64(m, 1H), 7.62–2.26 (m, 2H), 5.93 (s, 3H), 5.38-5.36 (m, 2H), 4.70-4.67 (m, 1H), 4.27-4.24 (m, 1H), 4.08-4.05 (m, 1H), 3.99–3.79 (m, 2H), 2.98-2.96 (m, 2H); MS (M+H$^+$) 302.18.

NBz-Leu-NH-(CH_2)$_4$-NH_2: white solid, yield 81 %; ^{1}H NMR (400 MHz, $CDCl_3$)δ 8.30 (d, *J* = 7.4 Hz, 2H), 7.99 (d, *J* = 7.6 Hz, 2H), 5.61 (s, 1H), 4.83 (s, 1H), 4.72–4.54 (m, 4H), 3.31-3.16 (m, 2H), 2.78-2.71 (m, 2H), 2.38–2.10 (m, 1H), 2.13–1.94 (m, 2H), 1.74-1.73 (m, 1H), 1.68–1.50 (m, 3H), 1.01 (d, *J* = 6.3 Hz, 6H); MS (M+H$^+$) 351.31.

Bz-Leu-NH-(CH_2)$_4$-NH_2: white solid, yield 61 %; ^{1}H NMR (400 MHz, $CDCl_3$)δ 8.01–7.81 (m, 2H), 7.58–7.36 (m, 3H), 7.31 (s, 1H), 5.81 (s, 1H), 5.07 (s, 3H), 4.42-4.40 (m, 1H), 3.23-3.19 (m, 2H), 2.79-2.64 (m, 2H), 2.03-1.95 (m, 2H), 1.94-1.88 (m, 1H), 1.83–1.57 (m, 4H), 1.2-1.00 (m, 6H); MS (M+H$^+$) 306.42.

1-NaftCO-Leu-NH-(CH_2)$_4$-NH_2: white solid, yield 89 %; ^{1}H NMR (400 MHz, $CDCl_3$)δ 8.92–8.72 (m, 1H), 8.02-7.98 (m, 1H), 7.97–7.81 (m, 2H), 7.70–7.45 (m, 3H), 4.83 (s, 1H), 4.70–4.52 (m, 4H), 3.28-3.19 (m, 2H), 2.77-2.67 (m, 2H), 2.00–1.75 (m, 4H), 1.65–1.48 (m, 3H), 1.12–0.95 (m, 6H); MS (M+H$^+$) 356.32.

NBz-Asn-NH-(CH_2)$_4$-NH_2: pale yellow solid, yield 78 %; ^{1}H NMR (400 MHz, $CDCl_3$)δ 8.32 (d, *J* = 7.4 Hz, 2H), 8.10 (d, *J* = 7.4 Hz, 2H), 5.77-5.76 (m, 5H), 5.66 (s, 1H), 5.25-5.26 (m, 1H), 5.05 (s, 1H), 3.41-3.05 (m, 3H), 2.87–2.65 (m, 3H), 1.97–1.82 (m, 2H), 1.72-1.66 (m, 2H); MS (M+H$^+$) 352.19.

Bz-Asn-NH-(CH_2)$_4$-NH_2: white solid, yield 69 %; ^{1}H NMR (400 MHz, $CDCl_3$)δ 7.87–7.70 (m, 2H), 7.50– 7.32 (m, 3H), 5.85 (s, 2H), 5.66 (s, 1H), 5.55 (s, 3H), 5.41-5.38 (m, 1H), 4.98 (s, 1H), 3.41-3.06 (m, 3H), 2.79–2.54 (m, 3H), 2.01-1.95 (m, 2H), 1.77-1.72 (m, 2H); MS (M+H$^+$) 307.12.

1-NaftCO-Asn-NH-(CH_2)$_4$-NH_2: white solid, yield 84 %; ^{1}H NMR (400 MHz, $CDCl_3$)δ 8.76–8.73 (m, 1H), 8.06–7.98 (m, 1H), 7.88–7.85 (m, 2H), 7.61–7.35 (m, 3H), 6.45 (s, 3H), 5.45 (s, 1H), 4.83 (s, 3H), 4.67-4.63 (m, 1H), 3.39–3.17 (m, 3H), 2.89–2.69 (m, 3H), 2.03-1.88 (m, 2H), 1.62-1.55 (m, 2H); MS (M+H$^+$) 357.24.

NBz-Phe-NH-(CH_2)$_4$-NH_2: pale yellow solid, yield 76 %; ^{1}H NMR (400 MHz, $CDCl_3$)δ 8.33 (d, *J* = 7.4 Hz, 2H), 8.12 (d, *J* = 7.4 Hz, 2H), 7.34–7.03 (m, 5H), 5.72 (s, 1H), 5.43 (s, 3H), 4.80-4.72 (m, 1H), 3.41–3.25 (m, 3H), 2.97-2.95 (m, 1H), 2.79-2.74 (m, 2H), 1.94-1.85 (m, 2H), 1.64–1.47 (m, 2H); MS (M+H$^+$) 385.27.

Bz-Phe-NH-(CH_2)$_4$-NH_2: white solid, yield 83 %; ^{1}H NMR (400 MHz, $CDCl_3$)δ 7.98–7.81 (m, 2H), 7.56-7.40 (m, 3H), 7.35 (s, 1H), 7.32–7.11 (m, 5H), 6.30–6.14 (s, 3H), 4.71 (s, 1H), 4.38 (s, 1H), 3.31-3.20 (m, 2H), 3.18-3.16 (m, 1H), 2.92-2.89 (m, 1H), 2.83–2.65 (m, 2H), 2.10–1.91 (m, 2H), 1.66–1.45 (m, 2H); MS (M+H$^+$) 340.42.

1-NaftCO-Phe-NH-(CH_2)$_4$-NH_2: white solid, yield 71 %; ^{1}H NMR (400 MHz, $CDCl_3$)δ 8.83–8.68 (m, 1H), 8.01-7.98 (m, 1H), 7.95–7.82 (m, 1H), 7.84–7.71 (m, 1H), 7.67–7.41 (m, 3H), 7.28–7.11 (m, 4H), 7.08–6.89 (m, 1H), 5.46 (s, 1H), 4.82-4.67 (m, 1H), 4.01 (s, 3H), 3.41-3.35 (m, 1H), 3.23-3.19 (m, 2H), 2.96-2.95 (m, 1H), 2.80-2.74 (m, 2H), 1.94-1.91 (m, 2H), 1.76–1.59 (m, 2H); MS ($M+H^+$) 390.28.

NBz-Ser-NH-(CH_2)$_4$-NH_2: white solid, yield 54 %; ^{1}H NMR (400 MHz, $CDCl_3$)δ 8.31 (d, *J* = 7.4 Hz, 2H), 8.14 (d, *J* = 7.4 Hz, 2H), 5.82 (s, 3H), 5.32 (s, 1H), 4.74 (s, 1H), 4.62-4.58 (m, 1H), 4.23-4.17 (m, 1H), 3.92-3.89 (m, 1H), 3.50-3.21 (m, 2H), 2.80-2.76 (m, 2H), 2.05-1.77 (m, 2H), 1.64-1.58 (m, 2H); MS ($M+H^+$) 325.33.

Bz-Ser-NH-(CH_2)$_4$-NH_2: white solid, yield 63 %; ^{1}H NMR (400 MHz, $CDCl_3$)δ 7.90–7.75 (m, 2H), 7.56–7.24 (m, 3H), 5.30-5.25 (m, 2H), 5.11 (s, 3H), 4.84-4.82 (m, 1H), 4.19-4.16 (m, 1H), 3.91-3.87 (m, 1H), 3.40-3.33 (m, 2H), 2.77-2.73 (m, 2H), 1.97-1.94 (m, 2H), 1.71-1.65 (m, 2H); MS ($M+H^+$) 280.34.

1-NaftCO-Ser-NH-(CH_2)$_4$-NH_2: white solid, yield 59 %; ^{1}H NMR (400 MHz, $CDCl_3$)δ 8.80–8.72 (m, 1H), 8.03–7.97 (m, 1H), 7.84-7.82 (m, 1H), 7.77-7.74 (m, 1H), 7.52-7.44 (m, 3H), 5.34 (s, 1H), 5.21 (s, 1H), 5.12 (s, 3H), 4.88-4.82 (m, 1H), 4.19-4.17 (m, 1H), 3.92-3.89 (m, 1H), 3.41-3.33 (m, 2H), 2.78-2.74 (m, 2H), 1.98-1.87 (m, 2H), 1.71-1.65 (m, 2H);MS ($M+H^+$) 330.14.

NBz-Leu-NH-cHx-NH_2: pale yellow solid, yield 89 %; ^{1}H NMR (400 MHz, $CDCl_3$)δ 8.32 (d, *J* = 7.4 Hz, 2H), 8.21 (d, *J* = 7.4 Hz, 2H), 6.69 (s, 1H), 5.87 (s, 1H), 4.69 (s, 3H), 4.47-4.43 (m, 1H), 3.67-3.62 (m, 1H), 3.56– 3.41 (m, 1H), 2.46–2.26 (m, 2H), 2.17–1.97 (m, 2H), 1.92–1.75 (m, 2H), 1.72–1.40 (m, 5H), 1.07–0.93 (m, 6H); MS ($M+H^+$) 377.18.

Bz-Leu-NH-cHx-NH_2: white solid, yield 75 %; ^{1}H NMR (400 MHz, $CDCl_3$)δ 7.88–7.73 (m, 2H), 7.56–7.29 (m, 3H), 6.67 (s, 1H), 5.21 (s, 1H), 5.06 (s, 3H), 4.42-4.39 (m, 1H), 3.31-3.28 (m, 1H), 3.08-3.02 (m, 1H), 2.24-2.16 (m, 2H), 2.12-2.04 (m, 2H), 1.87-1.80 (m, 1H), 1.72–1.50 (m, 4H), 1.41-1.28 (m, 2H), 1.03-0.99 (m, 6H); MS ($M+H^+$) 332.15.

1-NaftCO-Leu-NH-cHx-NH_2: white solid, yield 74 %; ^{1}H NMR (400 MHz, $CDCl_3$)δ 8.78–8.69 (m, 1H), 7.99-7.91 (m, 1H), 7.89-7.86 (m, 1H), 7.72-7.65 (m, 1H), 7.62–7.44 (m, 3H), 5.84 (s, 1H), 5.17 (s, 3H), 5.03 (t, *J* = 7.0 Hz, 1H), 4.92 (s, 1H), 3.97-3.93 (m, 1H), 3.52-3.45 (m, 1H), 2.42-2.36 (m, 2H), 2.24-2.14 (m, 2H), 2.08-2.01 (m, 2H), 1.82–1.67 (m, 1H), 1.65–1.47 (m, 4H), 1.07-1.01 (m, 6H); MS ($M+H^+$) 382.28.

NBz-Asn-NH-cHx-NH_2: white solid, yield 57 %; ^{1}H NMR (400 MHz, $CDCl_3$)δ 8.38 (s, 1H), 8.33 (d, *J* = 7.4 Hz, 2H), 8.18 (d, *J* = 7.6 Hz, 2H), 7.04 (s, 1H), 5.32 (s, 2H), 5.03 (s, 3H), 4.66–4.42 (m, 2H), 3.60–3.44 (m, 1H), 3.02-2.97 (m, 1H), 2.77-2.71 (m, 1H), 2.13-2.10 (m, 2H), 2.03–1.89 (m, 2H), 1.71-1.65 (m, 2H), 1.39-1.36 (m, 2H); MS ($M+H^+$) 378.32.

Bz-Asn-NH-cHx-NH_2: pale yellow, yield 51 %; ^{1}H NMR (400 MHz, $CDCl_3$)δ 8.45 (s, 1H), 7.98–7.82 (m, 2H), 7.68 (s, 1H), 7.58–7.22 (m, 3H), 5.14 (s, 2H), 5.09 (s, 3H), 4.45-4.39 (m, 1H), 3.35-3.37 (m, 1H), 3.07-3.01 (m, 1H), 2.95-2.89 (m, 1H), 2.58-2.54 (m, 1H), 2.38-2.34 (m, 2H), 2.20-2.16 (m, 2H), 1.75-1.71 (m, 4H); MS ($M+H^+$) 333.12.

NaftCO-Asn-NH-cHx-NH_2: white solid, yield 48 %; ^{1}H NMR (400 MHz, $CDCl_3$)δ 9.18–9.03 (m, 1H), 8.06-8.03 (m, 1H), 7.96-7.93 (m, 1H), 7.83-7.77 (m, 1H), 7.66–7.51 (m, 2H), 7.47-7.42 (m, 1H),

6.95 (s, 1H), 6.47 (s, 1H), 5.16 (s, 2H), 4.60–4.35 (m, 4H), 3.67–3.58 (m, 1H), 3.57–3.41 (m, 1H), 3.09-3.06 (m, 1H), 2.78-2.74 (m, 1H), 2.51–2.22 (m, 4H), 2.00–1.80 (m, 2H), 1.73–1.45 (m, 2H); MS ($M+H^+$) 383.71.

NBz-Phe-NH-cHx-NH2: white solid, yield 82 %; ^{1}H NMR (400 MHz, $CDCl_3$)) δ 8.34 (d, *J* = 7.6 Hz, 2H), 8.13 (d, *J* = 7.5 Hz, 2H), 7.32–6.97 (m, 5H), 6.71 (s, 1H), 5.21 (s, 1H), 5.03 (s, 3H), 4.75-4.67 (m, 1H), 3.76-3.68 (m, 1H), 3.54-3.47 (m, 1H), 3.44-3.37 (m, 1H), 3.01-2.89 (m, 1H), 2.86-2.81 (m, 2H), 1.88-1.76 (m, 4H), 1.23-1.16 (m, 2H); MS ($M+H^+$) 411.16.

Bz-Phe-NH-cHx-NH2: pale yellow, yield 63 %; ^{1}H NMR (400 MHz, $CDCl_3$)δ 7.96–7.64 (m, 2H), 7.60–7.36 (m, 3H), 7.30–6.98 (m, 5H), 5.61 (s, 1H), 5.09 (s, 3H), 4.82-4.75 (m, 2H), 3.93-3.87 (m, 1H), 3.47-3.44 (m, 1H), 3.02–2.75 (m, 2H), 2.11-1.98 (m, 2H), 1.75-1.68 (m, 2H), 1.59-1.46 (m, 2H), 1.24-1.18 (m, 2H); MS ($M+H^+$) 366.18.

1-NaftCO-Phe-NH-cHx-NH2: white solid, yield 65 %; ^{1}H NMR (400 MHz, $CDCl_3$)δ 8.62–8.50 (m, 1H), 7.99-7.96 (m, 1H), 7.91–7.84 (m, 1H), 7.73-7.65 (m, 1H), 7.60–7.45 (m, 3H), 7.35 7.07 (m, 5H), 5.72 (s, 1H), 5.27–5.14 (m, 4H), 5.09 (s, 1H), 4.34-4.26 (m, 1H), 3.48-3.45 (m, 1H), 3.23-3.18 (m, 1H), 2.94-2.88 (m, 1H), 2.64-2.55 (m, 2H), 2.38-2.30 (m, 2H), 1.95–1.67 (m, 4H); MS ($M+H^+$) 416.23.

NBz-Ser-NH-cHx-NH2: white solid, yield 49 %; ^{1}H NMR (400 MHz, $CDCl_3$)δ 8.32 (d, *J* = 7.4 Hz, 2H), 8.15 (d, *J* = 7.4 Hz, 2H), 7.62 (s, 1H), 5.13 (s, 3H), 4.64 (s, 1H), 4.22–3.99 (m, 3H), 3.87–3.72 (m, 1H), 3.58–3.35 (m, 1H), 2.41–2.21 (m, 2H), 2.17–2.01 (m, 2H), 2.04–1.92 (m, 2H), 1.58-1.56 (m, 2H); MS ($M+H^+$) 351.37.

Bz-Ser-NH-cHx-NH2: white solid, yield 50 %; ^{1}H NMR (400 MHz, $CDCl_3$)δ 7.89–7.70 (m, 2H), 7.57–7.32 (m, 3H), 6.23 (s, 1H), 5.20 (s, 1H), 5.05 (s, 3H), 4.38–4.15 (m, 2H), 3.91-3.88 (m, 1H), 3.32-.2.28 (m, 1H), 3.07-3.02 (m, 1H), 2.22-2.12 (m, 2H), 2.11-2.08 (m, 2H), 1.67-1.65 (m, 2H), 1.45-1.40 (m, 2H); MS ($M+H^+$) 306.22.

1-NaftCO-Ser-NH-cHx-NH2: pale yellow, yield 55 %; ^{1}H NMR (400 MHz, $CDCl_3$)δ 8.84–8.61 (m, 1H), 8.00-7.97 (m, 1H), 7.93–7.78 (m, 2H), 7.68–7.44 (m, 4H), 4.65-4.58 (m, 2H), 4.41–4.23 (m, 4H), 3.92-3.86 (m, 1H), 3.72-3.67 (m, 1H), 3.64 (s, 1H), 3.52-3.42 (m, 1H), 2.38–2.20 (m, 2H), 2.20–2.01 (m, 2H), 1.69–1.42 (m, 4H); MS ($M+H^+$) 356.38.

Analytical data of R'-Xaa-NH-R-NH-ClNafQ

HMB-Leu-NH-(CH_2)4-NH-ClNafQ (**17**): white solid; yield 57%;mp = 121-124 °C; $[\alpha]_D^{20}$ -11.5 (c=1, MeOH); ^{1}H NMR (400 MHz, $CDCl_3$)δ 8.26 (dd, *J* = 7.4, 1.4 Hz, 1H), 8.13 (dd, *J* = 7.5, 1.6 Hz, 1H), 7.80-7.76 (m, 1H), 7.70-7.66 (m, 1H), 7.30 (dd, *J* = 7.5, 1.4 Hz, 1H), 7.20 (s, 1H), 7.12 (t, *J* = 7.5 Hz, 1H), 6.89 (dd, *J* = 7.5, 1.4 Hz, 1H), 4.66 (s, 1H), 4.33-4.29 (m, 1H), 4.24 (s, 1H), 3.24-3.18 (m, 4H), 2.34 (s, 3H), 1.81–1.31 (m, 6H), 1.13–0.86 (m, 7H); MS (ESI): $[MH]^+$ = 526.21; HPLC (T_r) 15.40 min.

NBz-Leu-NH-(CH_2)4-NH-ClNafQ (**18**): white solid; yield 61%;mp = 115-117 °C; $[\alpha]_D^{20}$ -10.2 (c=1, MeOH); ^{1}H NMR (400 MHz, $CDCl_3$)δ 8.21-8.15 (m, 1H), 8.12–8.06 (m, 6H), 7.78-7.74 (m, 1H), 7.70-7.66 (m, 1H), 7.57 (s, 1H), 5.72 (s, 1H), 4.40-4.37 (m, 1H), 3.29 (m, 4H), 1.94-1.88 (m, 1H),

1.72–1.61 (m, 3H), 1.58–1.48 (m, 3H), 1.00– 092 (m, 6H); MS (ESI): $[MH]^+$ = 541.18; HPLC (T_r) 16.43 min.

Bz-Leu-NH-$(CH_2)_4$-NH-ClNafQ (**19**): white solid; yield 64%;mp = 120-123 °C; $[\alpha]_D^{20}$ -9.4 (c=1, MeOH); ^1H NMR (400 MHz, $CDCl_3$)δ 8.34–8.15 (m, 1H), 7.94–7.81 (m, 2H), 7.81–7.72 (m, 2H), 7.69-7.62 (m, 1H), 7.53–7.41 (m, 3H), 7.12 (s, 1H), 4.81 (s, 1H), 4.65 (s, 1H), 4.50-4.95 (m, 1H), 3.41–3.23 (m, 2H), 3.23–3.04 (m, 2H), 1.79-1.76 (m, 1H), 1.59–1.45 (m, 5H), 1.41-1.37 (m, 1H), 1.05-0.99 (m, 6H); MS (ESI): $[MH]^+$ = 496.20; HPLC (T_r) 16.10 min.

1-NaftCO-Leu-NH-$(CH_2)_4$-NH-ClNafQ (**20**): white solid; yield 51%;mp = 123-124 °C; $[\alpha]_D^{20}$ -13.5 (c=1, MeOH); ^1H NMR (400 MHz, $CDCl_3$)δ 9.34 (dd, *J* = 7.5, 1.6 Hz, 1H), 8.22 (dd, *J* = 7.4, 1.6 Hz, 1H), 8.14–8.04 (m, 2H), 7.97-7.94 (m, 1H), 7.81-7.79 (m, 1H), 7.73–7.41 (m, 6H), 5.41 (s, 1H), 4.24-4.16 (m, 2H), 3.34-3.27 (m, 1H), 3.28–3.11 (m, 3H), 1.78–1.65 (m, 3H), 1.63–1.54 (m, 2H), 1.49-1.45 (m, 1H), 1.26–1.01 (m, 1H), 0.83-0.76 (m, 6H); MS (ESI): $[MH]^+$ = 546.24; HPLC (T_r) 16.82 min.

HMB-Asn-NH-$(CH_2)_4$-NH-ClNafQ (**21**): white solid; yield 81%;mp = 145-148 °C; $[\alpha]_D^{20}$ -25.6 (c=1, MeOH); ^1H NMR (400 MHz, $CDCl_3$)δ 8.48 (s, 1H), 8.24-8.21 (m, 1H), 8.14-8.12 (m, 1H), 7.71-7.65 (m, 2H), 7.34 (s, 1H), 7.31–7.16 (m, 2H), 7.02–6.85 (m, 1H), 5.26 (s, 2H), 5.03 (s, 1H), 4.85-4.77 (m, 1H), 3.25-3.22 (m, 2H), 3.17-3.14 (m, 2H), 3.10-3.06 (m, 1H), 2.66-2.60 (m, 1H), 2.31 (s, 3H), 1.69–1.59 (m, 2H), 1.58–1.50 (m, 2H); MS (ESI): $[MH]^+$ = 527.11; HPLC (T_r) 12.36 min.

NBz-Asn-NH-$(CH_2)_4$-NH-ClNafQ (**22**): white solid; yield 59%;mp = 151-153 °C; $[\alpha]_D^{20}$ -18.1 (c=1, MeOH); ^1H NMR (400 MHz, $CDCl_3$)δ 8.37–8.30 (m, 1H), 8.26–8.08 (m, 3H), 8.00-7.89 (m, 2H), 7.81–7.71 (m, 2H), 7.65 (s, 1H), 7.13 (s, 1H), 5.45 (s, 2H), 5.38 (s, 1H), 4.66-4.61 (m, 1H), 3.30–3.20 (m, 4H), 2.81–2.76 (m, 1H), 1.66–1.61 (m, 2H), 1.44-1.38 (m, 2H); MS (ESI): $[MH]^+$ = 542.14; HPLC (T_r) 13.56 min.

Bz-Asn-NH-$(CH_2)_4$-NH-ClNafQ (**23**): white solid; yield 74%;mp = 142-145 °C; $[\alpha]_D^{20}$ -15.4 (c=1, MeOH); ^1H NMR (400 MHz, $CDCl_3$)δ 8.37–8.21 (m, 1H), 8.21–8.01 (m, 1H), 7.75–7.62 (m, 5H), 7.51–7.30 (m, 3H), 6.10 (s, 1H), 5.35 (s, 1H), 5.21 (s, 2H), 4.76-4.68 (m, 1H), 3.23-3.20 (m, 4H), 3.07–2.96 (m, 1H), 2.81–2.76 (m, 1H), 1.68–1.51 (m, 4H); MS (ESI): $[MH]^+$ = 498.24; HPLC (T_r) 13.31 min.

1-NaftCO-Asn-NH-$(CH_2)_4$-NH-ClNafQ (**24**): white solid; yield 62%;mp = 148-151 °C; $[\alpha]_D^{20}$ -22.6 (c=1, MeOH); ^1H NMR (400 MHz, $CDCl_3$)δ 8.41-4.38 (m, 1H), 8.25 – 8.07 (m, 3H), 7.99-7.95 (m, 1H), 7.86–7.76 (m, 2H), 7.75–7.62 (m, 3H), 7.58-7.53 (m, 1H), 7.49-7.45 (m, 1H), 6.62 (s, 1H), 5.18 (s, 1H), 5.10 (s, 2H), 4.97-4.88 (m, 1H), 3.35–3.16 (m, 4H), 3.16–3.02 (m, 1H), 2.64-2.62 (m, 1H), 1.64–1.46 (m, 4H); MS (ESI): $[MH]^+$ = 547.28; HPLC (T_r) 14.03 min.

HMB-Phe-NH-$(CH_2)_4$-NH-ClNafQ (**25**): white solid; yield 81%;mp = 118-120 °C; $[\alpha]_D^{20}$ -21.5 (c=1, MeOH); ^1H NMR (400 MHz, $CDCl_3$)δ 8.75 (s, 1H), 8.08-8.02 (m, 1H), 7.95-7.92 (m, 1H), 7.70-7.65 (m, 2H), 7.33-7.17 (m, 6H), 7.09-7.05 (m, 1H), 6.88-6.86 (m, 2H), 5.91 (s, 1H), 4.73-4.68 (m, 1H), 3.42-3.40 (m, 1H), 3.27–3.13 (m, 5H), 2.29 (s, 3H), 1.70–1.48 (m, 4H);MS (ESI): $[MH]^+$ = 560.17; HPLC (T_r) 14.70 min.

NBz-Phe-NH-$(CH_2)_4$-NH-ClNafQ (**26**): white solid; yield 71%;mp = 108-111 °C; $[\alpha]_D^{20}$ -18.2 (c=1, MeOH); ^1H NMR (400 MHz, $CDCl_3$)δ 8.31 (d, *J* = 7.4 Hz, 2H), 8.21–7.92 (m, 4H), 7.74–7.64 (m, 2H), 7.53 (s, 1H), 7.27–7.11 (m, 6H), 4.72-4.68 (m, 1H), 4.57 (s, 1H), 3.31–3.13 (m, 5H), 2.89-2.85 (m, 1H), 1.72–1.49 (m, 4H); MS (ESI): $[MH]^+$ = 575.12; HPLC (T_r) 15.88 min.

Bz-Phe-NH-$(CH_2)_4$-NH-ClNafQ (**27**): white solid; yield 61%;mp = 110-113 °C; $[\alpha]_D^{20}$ -13.4 (c=1, MeOH); ^{1}H NMR (400 MHz, $CDCl_3$)δ 8.18–8.03 (m, 2H), 7.92–7.86 (m, 2H), 7.73–7.66 (m, 2H), 7.49–7.37 (m, 3H), 7.35 (s, 1H), 7.31–7.22 (m, 3H), 7.22–7.16 (m, 2H), 7.05 (s, 1H), 4.78-4.70 (m, 1H), 4.67 (s, 1H), 3.33-3.27 (m, 1H), 3.24-3.18 (m, 4H), 2.93-2.89 (m, 1H), 1.62-1.55 (m, 2H), 1.50–1.42 (m, 2H); MS (ESI): $[MH]^+$ = 530.02; HPLC (T_r) 16.02 min.

1-NaftCO-Phe-NH-$(CH_2)_4$-NH-ClNafQ (**28**): white solid; yield 66%;mp = 115-117 °C; $[\alpha]_D^{20}$ -23.5 (c=1, MeOH); ^{1}H NMR (400 MHz, $CDCl_3$)δ 9.19 (s, 1H), 8.79–8.65 (m, 1H), 8.42 (s, 1H), 8.17–8.00 (m, 2H), 7.98–7.74 (m, 3H), 7.73–7.59 (m, 2H), 7.56–7.34 (m, 3H), 7.20–7.00 (m, 5H), 6.31 (s, 1H), 4.96-4.91 (m, 1H), 3.36–3.10 (m, 4H), 3.02-3.05 (m, 1H), 2.86-2.84 (m, 1H), 1.79–1.45 (m, 4H); MS (ESI): $[MH]^+$ = 580.19; HPLC (T_r) 16.39 min.

HMB-Ser-NH-$(CH_2)_4$-NH-ClNafQ (**29**): white solid; yield 44%;mp = 135-138 °C; $[\alpha]_D^{20}$ -35.6 (c=1, MeOH); ^{1}H NMR (400 MHz, $CDCl_3$)δ 8.28–8.02 (m, 2H), 7.81–7.61 (m, 2H), 7.47 (s, 1H), 7.30-7.27 (m, 1H), 7.14-7.11 (m, 1H), 6.89-6.81 (m, 1H), 5.62 (s, 1H), 5.58 (s, 1H), 4.52-4.44 (m, 1H), 4.29-4.25 (m, 1H), 4.09-4.01 (m, 1H), 3.40–3.12 (m, 4H), 1.70–1.51 (m, 4H); MS (ESI): $[MH]^+$ = 500.15; HPLC (T_r) 12.88 min.

NBz-Ser-NH-$(CH_2)_4$-NH-ClNafQ (**30**): white solid; yield 82%;mp = 130-133 °C; $[\alpha]_D^{20}$ -29.1 (c=1, MeOH); ^{1}H NMR (400 MHz, $CDCl_3$)δ 8.23–7.94 (m, 6H), 7.80-7.70 (m, 2H), 6.34 (s, 1H), 5.46 (s, 1H), 4.47 (s, 1H), 4.32-4.20 (m, 2H), 3.95-3.91 (m, 1H), 3.29-3.16 (m, 4H), 1.78–1.49 (m, 4H);MS (ESI): $[MH]^+$ = 515.17, HPLC (T_r) 13.99 min.

Bz-Ser-NH-$(CH_2)_4$-NH-ClNafQ (**31**): white solid; yield 58%;mp = 132-135 °C; $[\alpha]_D^{20}$ -20.4 (c=1, MeOH); ^{1}H NMR (400 MHz, $CDCl_3$)δ 8.24–8.10 (m, 2H), 7.88–7.71 (m, 5H), 7.49–7.34 (m, 3H), 5.25 (s, 1H), 5.15 (s, 1H), 4.48-4.46 (m, 1H), 4.18-4.14 (m, 1H), 3.90-3.85 (m, 1H), 3.28-3.17 (m, 4H), 1.72–1.55 (m, 2H), 1.48-1.42 (m, 2H); MS (ESI): $[MH]^+$ = 470.11; HPLC (T_r) 14.07 min.

1-NaftCO-Ser-NH-$(CH_2)_4$-NH-ClNafQ (**32**): white solid; yield 61%;mp = 139-141 °C; $[\alpha]_D^{20}$ -31.8 (c=1, MeOH); ^{1}H NMR (400 MHz, $CDCl_3$)δ 8.62 (dd, J = 7.5, 1.6 Hz, 1H), 8.19–8.10 (m, 3H), 7.96–7.85 (m, 2H), 7.82-7.79 (m, 1H), 7.68-7.65 (m, 2H), 7.53-7.43 (m, 3H), 5.90 (s, 1H), 5.03-4.99 (m, 2H), 4.24-4.21 (m, 1H), 4.04-4.01 (m, 1H), 3.45–3.35 (m, 1H), 3.30–3.11 (m, 3H), 1.70–1.55 (m, 4H);MS (ESI): $[MH]^+$ = 520.08; HPLC (T_r) 14.58 min.

HMB-Leu-NH-cHx-NH-ClNafQ (**33**): white solid; yield 69%;mp = 118-120 °C; $[\alpha]_D^{20}$ -9.7 (c=1, MeOH); ^{1}H NMR (400 MHz, $CDCl_3$)δ 8.27 – 8.00 (m, 2H), 7.71-7.67 (m, 2H), 7.35-7.29 (m, 2H), 7.13-7.07 (m, 1H), 6.96–6.75 (m, 2H), 4.91 (s, 1H), 4.59-4.51 (m, 1H), 3.77–3.45 (m, 2H), 2.36–2.08 (m, 5H), 1.97-1.92 (m, 1H), 1.89–1.75 (m, 1H), 1.76–1.55 (m, 5H), 1.39–1.15 (m, 2H), 0.96-0.94 (m, 6H);MS (ESI): $[MH]^+$ = 552.18; HPLC (T_r) 14.12 min.

NBz-Leu-NH-cHx-NH-ClNafQ (**34**): white solid; yield 54%;mp = 112-115 °C; $[\alpha]_D^{20}$ -8.2 (c=1, MeOH); ^{1}H NMR (400 MHz, $CDCl_3$)δ 8.27–8.18 (m, 3H), 8.18–8.11 (m, 1H), 8.08-8.01 (m, 2H), 7.95–7.59 (m, 2H), 7.21 (s, 1H), 5.75 (s, 1H), 5.23 (s, 1H), 4.86-4.82 (m, 1H), 3.87–3.59 (m, 1H), 3.56–3.36 (m, 1H), 2.22–1.91 (m, 4H), 1.8–1.59 (m, 6H), 1.61–1.45 (m, 1H), 1.04-1.00 (m, 6H); MS (ESI): $[MH]^+$ = 567.18; HPLC (T_r) 16.04 min.

Bz-Leu-NH-cHx-NH-ClNafQ (**35**): white solid; yield 56%;mp = 117-119 °C; $[\alpha]_D^{20}$ -7.6 (c=1, MeOH); ^{1}H NMR (400 MHz, $CDCl_3$)δ 8.14–8.01 (m, 2H), 7.90–7.80 (m, 2H), 7.77–7.59 (m, 3H), 7.53–7.30 (m, 3H), 6.20 (s, 1H), 4.82 (s, 1H), 4.57-4.51 (m, 1H), 3.76–3.49 (m, 2H), 2.24-2.17 (m, 2H), 2.04-1.88 (m, 2H), 1.91–1.74 (m, 2H), 1.75–1.49 (m, 5H), 1.08-0.98 (m, 6H); MS (ESI): $[MH]^+$ = 522.14; HPLC (T_r) 15.74 min.

1-NaftCO-Leu-NH-cHx-NH-ClNafQ (**36**): white solid; yield 81%;mp = 121-124 °C; $[\alpha]_D^{20}$ -11.8 (c=1, MeOH); ^{1}H NMR (400 MHz, $CDCl_3$)δ 8.90–8.64 (m, 1H), 8.20–8.04 (m, 2H), 8.04–7.93 (m, 1H), 7.92-7.87 (m, 1H), 7.76–7.59 (m, 3H), 7.53-7.48 (m, 2H), 7.43-7.37 (m, 1H), 6.05 (s, 1H), 5.60 (s, 1H), 5.30 (s, 1H), 4.53-4.44 (m, 1H), 4.09-4.01 (m, 1H), 3.89–3.66 (m, 1H), 2.11–1.95 (m, 1H), 1.97–1.66 (m, 7H), 1.62–1.40 (m, 3H), 1.06 (d, *J* = 6.3 Hz, 6H); MS (ESI): $[MH]^+$ = 572.10; HPLC (T_r) 16.40 min.

HMB-Asn-NH-cHx-NH-ClNafQ (**37**): white solid; yield 57%;mp = 144-145 °C; $[\alpha]_D^{20}$ -21.2 (c=1, MeOH); ^{1}H NMR (400 MHz, $CDCl_3$)δ 8.29–8.08 (m, 2H), 7.83–7.57 (m, 2H), 7.25–7.03 (m, 3H), 6.97 (s, 1H), 6.86-6.77 (m, 1H), 5.24 (s, 1H), 5.17 (s, 2H), 4.70-4.65 (m, 1H), 4.22–4.05 (m, 1H), 4.01–3.81 (m, 1H), 3.08-3.00 (m, 1H), 2.74-2.65 (m, 1H), 2.25-2.21 (m, 3H), 2.22–2.01 (m, 4H), 1.91–1.69 (m, 4H); MS (ESI): $[MH]^+$ = 553.19; HPLC (T_r) 13.34 min.

NBz-Asn-NH-cHx-NH-ClNafQ (**38**): white solid; yield 55%;mp = 148-151 °C; $[\alpha]_D^{20}$ -13.7 (c=1, MeOH); ^{1}H NMR (400 MHz, $CDCl_3$)δ 8.73 (s, 1H), 8.29–8.02 (m, 7H), 7.73-7.67 (m, 2H), 6.03 (s, 1H), 5.10 (s, 2H), 4.60-4.53 (m, 1H), 3.78-3.71 (m, 2H), 3.18-3.16 (m, 1H), 2.76-2.71 (m, 1H), 2.03-1.96 (m, 2H), 1.96-1.91 (m, 2H), 1.88-1.81 (m, 2H), 1.56-1.49 (m, 2H); MS (ESI): $[MH]^+$ = 568.16; HPLC (T_r) 14.66 min.

Bz-Asn-NH-cHx-NH-ClNafQ (**39**): white solid; yield 62%;mp = 134-135 °C; $[\alpha]_D^{20}$ -12.2 (c=1, MeOH); ^{1}H NMR (400 MHz, $CDCl_3$)δ 8.29–8.14 (m, 2H), 8.07 (s, 1H), 7.87-7.81 (m, 3H), 7.80–7.59 (m, 2H), 7.56–7.34 (m, 3H), 7.24 (s, 1H), 4.34 (s, 2H), 4.27–4.08 (m, 2H), 4.10–3.85 (m, 1H), 2.96-2.93 (m, 1H), 2.74-2.69 (m, 1H), 2.20–2.04 (m, 2H), 2.03–1.86 (m, 4H), 1.74–1.56 (m, 2H); MS (ESI): $[MH]^+$ = 523.27; HPLC (T_r) 14.44 min.

1-NaftCO-Asn-NH-cHx-NH-ClNafQ (**40**): white solid; yield 68%;mp = 151-152 °C; $[\alpha]_D^{20}$ -18.8 (c=1, MeOH); ^{1}H NMR (400 MHz, $CDCl_3$)δ 9.04–8.47 (m, 1H), 8.20–8.07 (m, 2H), 8.08–7.93 (m, 2H), 7.70-7.66 (m, 4H), 7.57-7.51 (m, 1H), 7.44-7.41 (m, 1H), 7.30 (s, 1H), 6.43 (s, 1H), 5.50 (s, 2H), 4.61-4.56 (m, 1H), 4.08–3.86 (m, 1H), 3.72 (s, 1H), 3.13-3.09 (m, 1H), 2.95–2.69 (m, 2H), 2.06–1.83 (m, 2H), 1.72-1.67 (m, 2H), 1.64–1.43 (m, 2H), 1.18-1.11 (m, 2H); MS (ESI): $[MH]^+$ = 573.21; HPLC (T_r) 15.70 min.

HMB-Phe-NH-cHx-NH-ClNafQ (**41**): white solid; yield 77%;mp = 120-121 °C; $[\alpha]_D^{20}$ -11.4(c=1, MeOH); ^{1}H NMR (400 MHz, $CDCl_3$)δ 8.20–7.98 (m, 2H), 7.77–7.56 (m, 3H), 7.31-2.25 (m, 1H), 7.25–7.02 (m, 6H), 6.89-6.83 (m, 1H), 5.40 (s, 1H), 5.24-5.17 (m, 1H), 5.14 (s, 1H), 3.89–3.67 (m, 2H), 3.34-3.29 (m, 1H), 3.00-2.93 (m, 1H), 2.31 (s, 3H), 2.07–1.80 (m, 6H), 1.76–1.62 (m, 2H); MS (ESI): $[MH]^+$ = 586.15;HPLC (T_r) 14.76 min.

NBz-Phe-NH-cHx-NH-ClNafQ (**42**): white solid; yield 59%;mp = 115-117 °C; $[\alpha]_D^{20}$ -9.4 (c=1, MeOH); ^{1}H NMR (400 MHz, $CDCl_3$)δ 8.29 (d, *J* = 7.4 Hz, 2H), 8.18–8.00 (m, 4H), 7.83–7.45 (m, 3H), 7.32–6.92 (m, 5H), 5.83 (s, 1H), 5.21-5.17 (m, 1H), 5.09 (s, 1H), 4.74-4.65 (m, 1H), 3.99-3.87 (m, 1H), 3.18-3.15 (m, 1H), 2.96-2.94 (m, 1H), 2.36-2.31 (m, 4H), 2.11-2.02 (m, 2H), 1.59-1.51 (m, 2H); MS (ESI): $[MH]^+$ = 601.17; HPLC (T_r) 16.88 min.

Bz-Phe-NH-cHx-NH-ClNafQ (**43**): white solid; yield 71%;mp = 128-129 °C; $[\alpha]_D^{20}$ -10.6(c=1, MeOH); ^{1}H NMR (400 MHz, $CDCl_3$)δ 8.09–7.98 (m, 2H), 7.88–7.79 (m, 2H), 7.74–7.63 (m, 3H), 7.50–7.31 (m, 3H), 7.29–7.02 (m, 5H), 6.24 (s, 1H), 5.30-5.25 (m, 1H), 5.13 (s, 1H), 4.58-4.51 (m, 1H), 4.43-4.37 (m, 1H), 3.19-3.15 (m, 1H), 2.96-2.89 (m, 1H), 2.33-2.27 (m, 2H), 2.24-2.16 (m, 2H), 1.91-1.83 (m, 2H), 1.70-1.65 (m, 2H); MS (ESI): $[MH]^+$ = 556.20; HPLC (T_r) 16.70 min.

1-NaftCO-Phe-NH-cHx-NH-ClNafQ (**44**): white solid; yield 78%;mp = 133-134 °C; $[\alpha]_D^{20}$ -15.4(c=1, MeOH); ^{1}H NMR (400 MHz, $CDCl_3$)δ 8.60–8.40 (m, 1H), 8.07–7.97 (m, 2H), 7.92-7.88 (m, 1H), 7.85–7.71 (m, 2H), 7.74–7.56 (m, 3H), 7.60–7.39 (m, 3H), 7.32–7.05 (m, 5H), 5.68 (s, 1H), 5.26-5.15 (m, 1H), 5.19 (s, 1H), 4.92-4.89 (m, 1H), 4.46-4.39 (m, 1H), 3.22-3.19 (m, 1H), 2.96-2.91 (m, 1H), 2.39-2.31 (m, 2H), 2.29-2.13 (m, 2H), 2.02-1.98 (m, 2H), 1.76-1.70 (m, 2H); MS (ESI): $[MH]^+$ = 606.11; HPLC (T_r) 17.10 min.

HMB-Ser-NH-cHx-NH-ClNafQ (**45**): white solid; yield 71%;mp = 142-146 °C; $[\alpha]_D^{20}$ -23.4 (c=1, MeOH); ^{1}H NMR (400 MHz, $CDCl_3$)δ 8.32–8.12 (m, 2H), 7.80–7.60 (m, 2H), 7.32-7.27 (m, 1H), 7.14-7.08 (m, 1H), 6.94–6.68 (m, 2H), 5.73 (s, 1H), 5.31 (s, 1H), 4.62-4.54 (m, 1H), 4.19-4.13 (m, 1H), 3.87-3.81 (m, 3H), 2.36 (s, 3H), 2.21–1.96 (m, 4H), 1.94–1.73 (m, 4H); MS (ESI): $[MH]^+$ = 526.16; HPLC (T_r) 11.6 min.

NBz-Ser-NH-cHx-NH-ClNafQ (**46**): white solid; yield 54%;mp = 147-150 °C; $[\alpha]_D^{20}$ -16.5 (c=1, MeOH); ^{1}H NMR (400 MHz, $CDCl_3$)δ 8.17–8.03 (m, 4H), 7.98–7.80 (m, 3H), 7.70-7.65 (m, 2H), 5.17 (s, 1H), 5.09 (s, 1H), 4.55-4.49 (m, 1H), 4.18-4.12 (m, 1H), 4.10–4.01 (m, 1H), 3.94-3.91 (m, 1H), 3.90–3.71 (m, 1H), 2.14–1.80 (m, 6H), 1.72–1.52 (m, 2H); MS (ESI): $[MH]^+$ = 541.22; HPLC (T_r) 13.74 min.

Bz-Ser-NH-cHx-NH-ClNafQ (**47**): white solid; yield 83%;mp = 138-141 °C; $[\alpha]_D^{20}$ -13.8 (c=1, MeOH); ^{1}H NMR (400 MHz, $CDCl_3$)δ 8.27–8.10 (m, 2H), 7.95–7.82 (m, 2H), 7.78–7.63 (m, 2H), 7.56–7.30 (m, 4H), 6.56 (s, 1H), 5.04 (s, 1H), 4.54-4.49 (m, 1H), 4.24-4.17 (m, 1H), 3.87-3.81 (m, 1H), 3.71–3.55 (m, 1H), 3.43-3.33 (m, 1H), 2.37-2.31 (m, 2H), 2.16-2.11 (m, 2H), 1.93-1.84 (m, 2H), 1.75-1.70 (m, 2H);MS (ESI): $[MH]^+$ = 496.16; HPLC (T_r) 13.41 min.

1-NaftCO-Ser-NH-cHx-NH-ClNafQ (**48**): white solid; yield 66%;mp = 145-148 °C; $[\alpha]_D^{20}$ -20.7 (c=1, MeOH); ^{1}H NMR (400 MHz, $CDCl_3$)δ 8.97 (s, 1H), 8.75 (dd, *J* = 7.5, 1.6 Hz, 1H), 8.21–8.05 (m, 3H), 7.87–7.77 (m, 2H), 7.76–7.62 (m, 3H), 7.58-7.51 (m, 1H), 7.48-7.40 (m, 1H), 5.29 (s, 1H), 4.25-4.21 (m, 1H), 4.14-4.07 (m, 1H), 4.01–3.92 (m, 1H), 3.92–3.83 (m, 1H), 3.38–3.22 (m, 1H), 3.17 (s, 1H), 2.17-2.11 (m, 2H), 1.88-1.76 (m, 2H), 1.53-1.46 (m, 2H), 1.30-1.24 (m, 2H); MS (ESI): $[MH]^+$ = 546.13; HPLC (T_r) 14.30 min.
